# Optimizing separations in online comprehensive two‐dimensional liquid chromatography

**DOI:** 10.1002/jssc.201700863

**Published:** 2017-11-23

**Authors:** Bob W.J. Pirok, Andrea F.G. Gargano, Peter J. Schoenmakers

**Affiliations:** ^1^ University of Amsterdam Analytical‐Chemistry Group van ’t Hoff Institute for Molecular Sciences Amsterdam The Netherlands; ^2^ TI‐COAST Science Park Amsterdam The Netherlands; ^3^ Vrije Universiteit Amsterdam Department of Bioanalytical Chemistry Amsterdam Institute for Molecules Medicines and Systems Amsterdam The Netherlands

**Keywords:** comprehensive two‐dimensional liquid chromatography, method development, supercritical fluid chromatography

## Abstract

Online comprehensive two‐dimensional liquid chromatography has become an attractive option for the analysis of complex nonvolatile samples found in various fields (e.g. environmental studies, food, life, and polymer sciences). Two‐dimensional liquid chromatography complements the highly popular hyphenated systems that combine liquid chromatography with mass spectrometry. Two‐dimensional liquid chromatography is also applied to the analysis of samples that are not compatible with mass spectrometry (e.g. high‐molecular‐weight polymers), providing important information on the distribution of the sample components along chemical dimensions (molecular weight, charge, lipophilicity, stereochemistry, etc.). Also, in comparison with conventional one‐dimensional liquid chromatography, two‐dimensional liquid chromatography provides a greater separation power (peak capacity). Because of the additional selectivity and higher peak capacity, the combination of two‐dimensional liquid chromatography with mass spectrometry allows for simpler mixtures of compounds to be introduced in the ion source at any given time, improving quantitative analysis by reducing matrix effects. In this review, we summarize the rationale and principles of two‐dimensional liquid chromatography experiments, describe advantages and disadvantages of combining different selectivities and discuss strategies to improve the quality of two‐dimensional liquid chromatography separations.

**Abbreviations**
^1^D: first dimension
^2^D: second dimension
AEX: anion‐exchange chromatography
AfC: affinity chromatography
AgLC: argentation liquid chromatography
CAD: charged‐aerosol detector
CEX: cation‐exchange chromatography
DF: dilution factor
GC × GC: comprehensive two‐dimensional gas chromatography
HDC: hydrodynamic chromatography
HIC: hydrophobic‐interaction chromatography
LC × LC: comprehensive two‐dimensional liquid chromatography
LCCC: liquid chromatography at critical conditions
NPLC: normal‐phase liquid chromatography
PO: Pareto optimality
SAX: strong anion‐exchange chromatography
SCX: strong cation‐exchange chromatography
SFC × SFC: comprehensive two‐dimensional supercritical fluid chromatography
WAX: weak anion‐exchange chromatography
WCX: weak cation‐exchange chromatography

## 1 INTRODUCTION

LC is one of the most important and pervasive tools in the repertoire of analytical chemists. This is because of (i) the large variety of components found as building blocks or final products in nature and industrial production, (ii) the resulting complex, soluble mixtures of nonvolatile analytes, (iii) the remarkable selectivity and versatility of LC, (iv) the robustness and reliability of the technique, (v) the possibility of rigorous quantification, and (vi) the many ways in which LC can be combined with various detectors and other analytical instruments, including MS. One way to express the resolving power is the peak capacity of the separation system, i.e., the number of peaks that can be resolved with a given, equal resolution 1.

**Figure 1:**
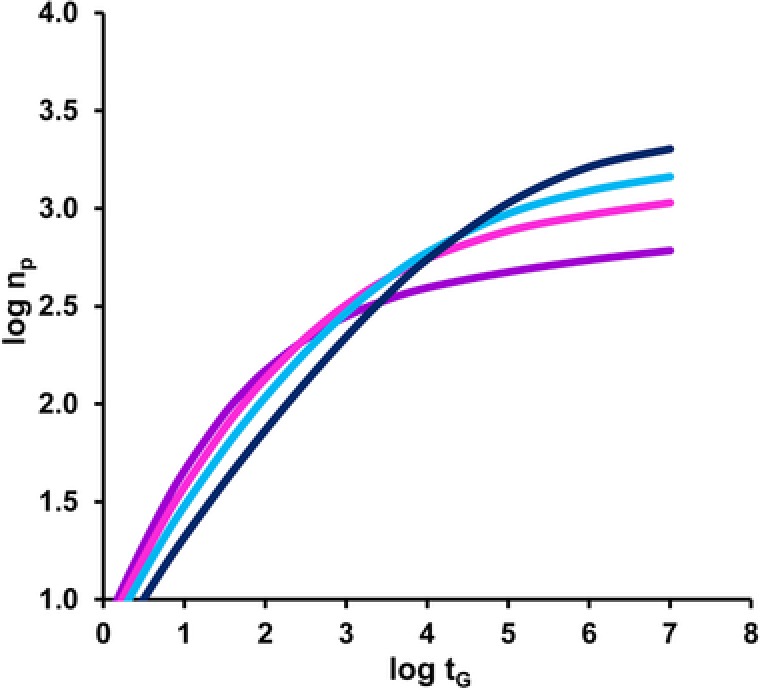
Estimated maximum attainable peak capacity as a function of the gradient time(s) for HPLC separation of peptides, following the procedure described in 12. *T* = 40°C, *ΔP* = 40 MPa, starting composition 5% acetonitrile in water. Lines (from top to bottom in top right corner) represent 5 μm (black), 3 μm (blue), 2 μm (pink), and 1 μm (purple) particles

In recent years, the attainable performance of 1D‐LC has been enhanced in a number of ways, for example by using long columns operated under ultra‐high pressure conditions (UHPLC) 2, highly efficient core–shell particles 3, 4, 5, monolithic stationary phases 6, or elevated temperatures 7. These developments have led to peak capacities up to about a thousand 8, 9, 10, as illustrated in Fig. 1.

However, Davis and Giddings have shown that the number of components that will statistically be separated as singular peaks (*p*) remains far below the theoretical peak capacity ($n_{c}$) 11.

$$\ln p=\ln m-\frac{m}{n_{c}}\;$$

For a sample containing m=1000 components, a theoretical peak capacity of 1000 can be expected to yield complete separation of samples containing no more than 370 peaks. To separate 95% of the peaks, a peak capacity of 20 000 would be required, i.e. 20‐fold excess. While the introduction of sub‐ 2 μm particles and the associated higher efficiencies represent a welcome addition to the toolkit of the chromatographer, their main advantage is in fast, rather than high‐resolution (high‐peak‐capacity) separations (see Fig. 1) 12. Samples containing more than 50 analytes are thus expected to result in (fully or partially) overlapping peaks even in the most efficient (and lengthy) 1D‐LC separations. The analyst may use experience and knowledge, possibly augmented with smart algorithms, to beat statistics by optimizing the chromatographic selectivity. Alternatively, the analyst may seek refuge in hyphenation with MS. LC–MS is a powerful technique, but accurate deconvolution of peaks requires that (i) the analytes and the mobile phase are compatible with the mass spectrometer, (ii) all analytes are present at similar (low) concentrations, (iii) only a few analytes are introduced into the ion source simultaneously, and (iv) the analytes are not isobaric (or at least yield sufficiently different mass or MS/MS spectra). LC can be seen as an online sample‐preparation technique to introduce simplified mixtures into the ion source, an inlet capable of separating sample components in the time domain. Mass spectrometrists share the desire of chromatographers for more resolving power and higher peak capacities, traits potentially offered by 2D‐LC.

In 2D‐LC, analyte‐containing fractions of the effluent of the first‐dimension (^1^D) column are transferred to the second‐dimension (^2^D) column and subjected to an additional separation. Transfer can be accomplished offline or online (using a valve interface). When one or a few distinct fractions are collected, we speak of heart‐cut 2D‐LC. In case of one fraction, the time spent on the ^2^D separation may be long. If a large number of fractions are transferred, so as to (largely) maintain the separation achieved in the first dimension (^1^D), we speak of comprehensive 2D‐LC (LC × LC) 13, 14. An intermediate option is to transfer (a) series of fractions across one or more interesting regions in the ^1^D chromatogram 15. This is known as selective comprehensive 2D‐LC. This review will be limited to method development for and optimization of LC × LC separations. However similar principles for optimization and column selection may be applied also to other 2D‐LC approaches.

The success of LC × LC can mainly be attributed to two factors, viz. the combination of two liquid separations with (very) different selectivities (ideally targeting different sample dimensions) and the greatly increased peak capacity (thanks to a reduction of the average peak widths to a few seconds), without an accompanying increasing in analysis time 16. Unfortunately, the complexity of method development does increase significantly, due to several reasons. First, an LC × LC method requires a decision on the two separation dimensions. These must be carefully considered, as they need to (i) be selective with respect to the sample dimensions, (ii) be compatible with each other in terms of mobile‐phase solvents, (iii) have one dimension that can be fast (e.g., <2 min), and (iv) be compatible with the detector. LC offers a number of choices for potentially very different retention mechanisms, including RPLC, normal‐phase LC (NPLC), HILIC SEC, IEC, ion‐pairing chromatography, hydrophobic‐interaction chromatography (HIC), and more.

To efficiently utilize the increased resolving power in LC × LC, the two separation dimensions must be sufficiently different (“orthogonal”, 17). This should aid the contemporary analyst, who is often confronted with “multidimensional samples”. The sample dimensionality theorem of Giddings 18 defines the dimensionality of the sample as the number of molecular blocks required to describe the constituting molecules. For example, a mixture of surfactants may feature variation in the length of a hydrophobic chain and variation in the number of charged end‐groups, presenting us with two sample dimensions. Giddings explained that the number of separation dimensions should ideally meet the number of sample dimensions. In the above case, this would translate into a hydrophobicity‐based separation, such as RPLC, and a charged‐based separation, such as IEC.

The requirement of orthogonal separation mechanisms in the two dimensions underlines the selectivity advantage of LC × LC. The multi‐modal selectivity allows structured (“group‐type”) separations if the retention mechanisms are carefully selected. In a group‐type separation, the different compound classes will elute in different areas of the 2D separation space.

Undesirable effects due to incompatibility of the mobile phases of the two dimensions or incompatibility of the ^2^D eluent with the detector have to be sensibly evaluated. The large choice of separation mechanisms implies an even greater number of possible combinations and this results in a potentially very large number of compatibility problems.

Setting up a complete LC × LC method requires a conscious consideration of a number of (sample‐independent) physical parameters, such as column dimensions, particle sizes, injection volumes, flow rates, and modulation time.

Optimal values of a number of “chemical” parameters that affect the selectivity must be sought. This typically concerns the mobile‐phase composition as a function of time (i.e., isocratic elution or multistep gradient programs) in both dimensions, possibly augmented by changes in other parameters, such as temperature, pH, or ionic strength of the mobile phase.

Thus, comprehensive method development involves a number of sample‐independent (physical) system parameters and an intricate tailoring of the sample‐dependent parameters that affect retention and selectivity. The best set of all parameters is considered the optimal method and optimization in chromatography entails the pursuit of this optimum. However, with the advent of state‐of‐the‐art instrumentation for LC × LC, the number of options to realize and optimize LC × LC separations has increased dramatically. Method development for LC × LC is lengthy and cumbersome and developing methods that make full use of the possibilities of the instrument (e.g., gradually shifting gradient parameters in the ^2^D) is barely possible to date. The challenge of rigorously optimizing LC × LC separations is complicated. The scope of most (if not all) methods remains solving an analytical question and this is what optimization processes should target. It is essential to overcome the method‐development bottleneck if sophisticated LC × LC systems are to be utilized to their full potential in an efficient manner. The cumbersome method‐development process in LC × LC can be divided in three steps (i) the (initial) design of the instrumental setup, (ii) the choice of the correct selectivities, and (iii) the optimization of the separation. In this review, each of these aspects is addressed and a number of guidelines will be presented for LC × LC method development and optimization.

## 2 FROM 1D TO 1D × 1D

It is good to realize that, ultimately, all LC × LC methods are the product of two 1D‐LC experiments. Consequently, it is necessary to start with establishing the two individual 1D separations when developing an LC × LC method. This will not only provide valuable insight in the retention behavior of the analyte mixture in relation to the distinct retention mechanisms in question, but will also ensure that the effects of the chromatographic conditions on the analyte mixture are understood. Therefore, the first key decision to make is which separation modes to use for each of the dimensions.

However, in anticipation of combining the two individual separation dimensions, the 2D separation system as a whole must be taken into account. While the choice of two orthogonal selectivities is of paramount importance, there are several other fundamental points to consider. In this section, we will address these key points before moving on to the choice of selectivities.

### 2.1 Elution Modes and their impact on 2D separations

#### 2.1.1 Isocratic versus gradient elution

In most cases, complex samples contain analytes with a wide range of retention factors. The use of isocratic elution, where the chemical parameters that affect retention and selectivity (e.g., composition of the mobile phase), are kept constant during the experiment, may not yield satisfactory elution of all compounds. As an example, keeping the fraction of strong solvent of the mobile phase constant during an RPLC separation imposes severe constraints. A low fraction of organic modifier may result in the efficient elution of weakly retained compounds, but strongly retained compounds will elute very late, leading to unreasonable analysis times and severe dilution of the analytes. On the other hand, favoring the elution of strongly retained analytes through a high concentration of organic modifier will result in poor resolution for weakly retained components, which are likely to elute close to the dead volume.

It is therefore not surprising that gradient elution is predominantly applied in LC × LC separations. In gradient elution, the chromatographic conditions (chemical parameters) are altered during the experiment. Depending on the selected retention mechanism, different types of gradients and gradient programs are applied. In RPLC, the organic‐modifier fraction is typically changed, whereas in IEX the ionic strength or pH is gradually altered. The gradient program can also be tailored to the separation, with a linear gradient most commonly applied. Some separations may require different gradient programs. For example, the exponential dependence of the retention in IEX on the salt concentration sometimes leads to the use of multistep gradients, where the salt concentration is increased following several consecutive linear gradient segments with increasing steepness (i.e., increasing slopes).

Major advantages of the use of gradient elution include (i) the coverage of a broader retention range, (ii) more even spacing of analyte peaks, and (iii) similar peak widths for most analyte bands, which promotes equal sampling of all analyte bands across the ^1^D. Because the bandwidths of late‐eluting peaks are reduced in gradient elution, a higher peak capacity can be obtained within a given time 19.

Despite these major advantages of gradients, application of gradient elution in the second dimension (^2^D) is not straightforward. Time must be allocated for postgradient column equilibration, reducing the time available for separation and, thus, the peak capacity obtained in the ^2^D. While in the case of RPLC, a single column volume may suffice for repeatable separation performance along different modulations 20, 21, this is not necessarily the case for other retention mechanisms. For IEX, extensive regeneration and reequilibration severely restrict the possibilities of using this retention mechanism in the ^2^D.

However, the application of a gradient in the ^2^D does facilitate the timely elution of strongly retained components. It also helps ensuring that all analytes elute from the column, so as to avoid wrap‐around effects, where analytes do not elute from the ^2^D column within the modulation time, but elute during the next modulation(s). Application of a gradient in the ^2^D requires a trade‐off between equilibration time and analysis time, but in most cases it is preferred over isocratic elution.

#### 2.1.2 Tailored second‐dimension gradients

In most LC × LC methods, the ^2^D gradient program is identical for all modulations. Such gradients typicaly span a broad range in composition in combination with a steep slope to accommodate the entire range of fractions form the ^1^D separation. However, with the ^1^D mechanism ideally orthogonal to the ^2^D, we can assume that each fraction presented to the ^2^D contains a different sample composition. Arguably, if we assume the sample composition to be largely constant (e.g. in a QC situation), each modulation may be treated as a unique, different 1D experiment, ideally requiring its own optimized gradient program.

**Figure 2:**
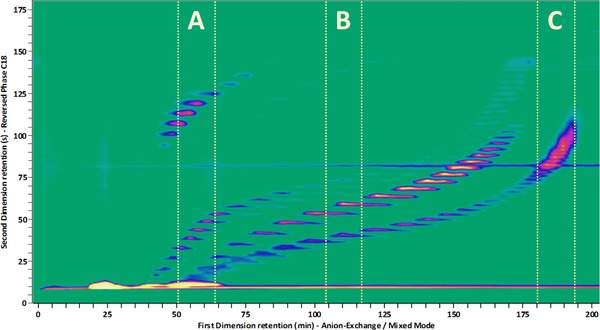
Separation of industrial surfactant mixture by mixed‐mode anion‐exchange chromatography and RPLC using a charged‐aerosol detector 22 (see Supporting Information Section S1 for the analysis method details used to record this data)

An example is shown in Fig. 2 where the separation of industrial surfactants by mixed‐mode strong IEX (AEX/CEX/RPLC) in the anion‐exchange mode and RPLC (AEX/CEX/RPLC × RPLC) using a charged‐aerosol detector is displayed 22 (see Supporting Information section S1 for the analysis method details). The separation appears satisfactory, with the relevant oligomeric series separated in a group‐type pattern. However, we also observe an ostensibly poor use of the separation space. In the modulations of section A of the chromatogram, the separation of weakly retained analytes is insufficient. A tailored gradient with an early shallow segment would be more favorable. Conversely, the separation in section B is satisfactory within the first half of the gradient, neatly resolving the analytes, but the latter half of the separation space is entirely unutilized. Strikingly, in section C all the peaks cluster near the center of the employed gradient and a more shallow gradient around this elution composition would significantly improve the resolution and promote the efficient use of the separation space.

**Figure 3:**
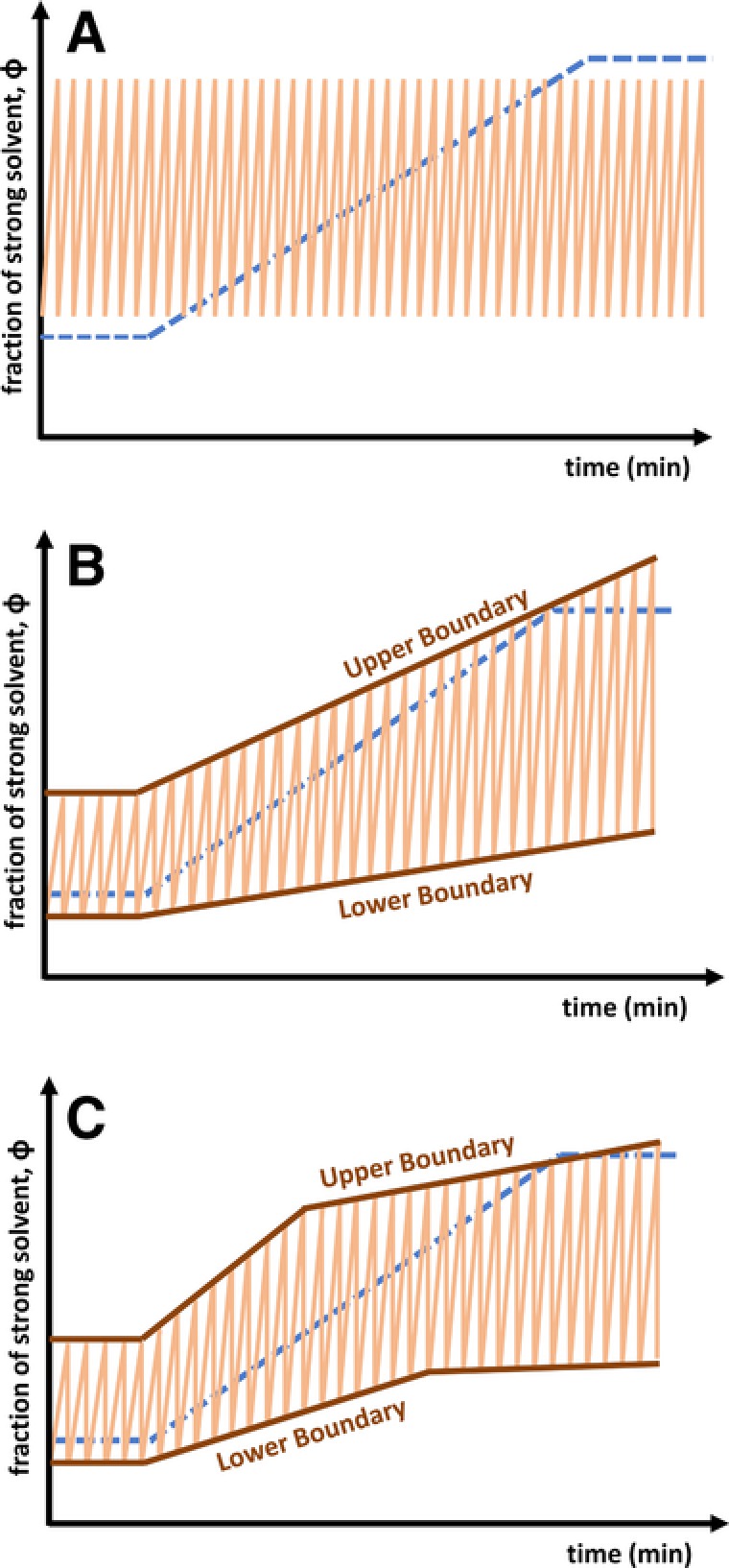
Overview of useful ^2^D elution programs. A, full‐in‐fraction; B, shifting; and C, multi‐segment shifting

Of course, developing a tailored gradient program for each individual modulation will lead to very long method‐development times. Fortunately, state‐of‐the‐art equipment allows the use of varying or shifting ^2^D gradients along the duration of the LC × LC experiment. In Fig. 3 several gradient assemblies are shown. The most commonly applied assembly pattern has been called “full‐in‐fraction” 23 (Fig. 3A). The broad gradient range guarantees good coverage of retention factors at the cost of limited resolution and possibly long equilibration times. Figure 3B displays a typical example of a (continuously) shifting gradient 24. In practice, there may be some correlation between the two retention mechanisms used in the separation dimensions. As a result, the ^2^D gradient assembly may relate to the ^1^D gradient. The degree of relation depends on the correlation between the two dimensions. This is true in comprehensive 2D GC (GC × GC), where the ^2^D temperature is closely related to that in the ^1^D, minimizing the effect of analyte volatility on the ^2^D retention time 25. In LC, certain RPLC × RPLC separation systems present similarly good examples 26.

Contemporary equipment allows the end‐user to define arbitrary boundaries between which the ^2^D gradients operate (Fig. 3C), thus allowing a large number of different “shifting” gradients. While seemingly complicated, an example of an effective, yet simple design of the boundaries is provided in Supporting Information Section S2. Of course, the design of gradient assemblies requires the user to take into account possible variations in the sample mixtures for which the method is developed. In establishing a given gradient assembly using the information from a specific sample mixture, possible unknown and unexpected analytes, which may manifest themselves in other samples, are ignored.

### 2.2 How to couple two separation dimensions in LC × LC: Modulation interface

In LC × LC, the columns are coupled by means of a transfer interface, generally called modulator. The modulator is typically a high‐pressure (ten‐ or eight‐port, two‐position) switching‐valve, which fractionates the effluent of the ^1^D using two installed loops. While one of the loops is being filled, the other loop is used as injector for the second‐separation dimension. Regular valve switches control the filling and injection cycles between the two loops, allowing to simultaneously sample and analyze ^1^D fractions. Alternative modulation approaches have been described. For further reading we suggest two reviews on this subject 27, 28.

**Figure 4:**
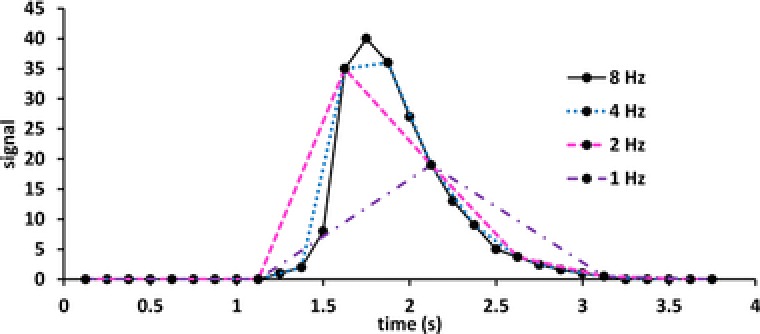
Illustration of the effect of undersampling on the reconstruction of the ^1^D chromatogram. Reconstruction at 8 Hz (solid black line), 4 Hz (dotted blue line), 2 Hz (pink dashed line), and 1 Hz (purple dash‐dotted line)

One of the most critical factors in 1D or 2D chromatography is the rate at which a peak is sampled. In 1D‐LC this corresponds to the detector sampling frequency (limited, for example, by the scanning rate of the MS). In LC × LC, the sampling rate is determined by the analysis time in the ^2^D. The analogy between detection in 1D‐LC and the modulation time in LC × LC is helpful for a quantitative description of the effects of a low sampling rate (“undersampling”) 29, 30, 31. The significance of the sampling rate is illustrated in Fig. 4

**Figure 5:**
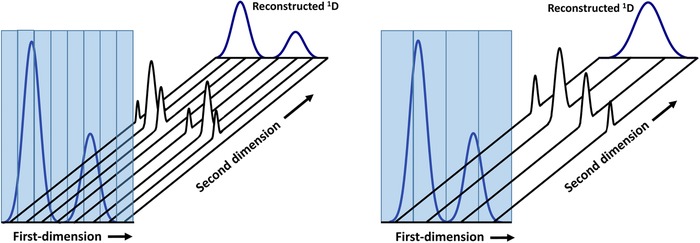
Schematic illustration of undersampling in comprehensive 2D chromatography

Undersampling results in loss of ^1^D resolution and quantitative and qualitative information. In Fig. 5, the ^1^D chromatogram (shaded box) is sampled at high (left) or low (right) frequency by a ^2^D separation in which the analytes present the same retention. When the ^1^D chromatogram is reconstructed from the 2D data, the resolution is maintained on the left side, but lost completely in case of undersampling as is seen on the right side.

Ideally, to avoid remixing of components already resolved from the ^1^D separation, while making the best possible use of the time available for separation, between two and three fraction should be transferred per ^1^D peak 31, 32, 33. This restricts the cycle time for the ^2^D separation.

### 2.3 Physical Parameters

For a 2D‐LC method to be considered comprehensive, it must meet the premise that the entire ^1^D effluent is subjected to the ^2^D separation. This means that in principle (i) the ^2^D analysis time must be sufficiently short to allow processing of the incoming fractions from the ^1^D, (ii) the ^1^D flow rate must match the requirements of the modulator and of the ^2^D separation, and (iii) the sampling loops must be able to store all incoming ^1^D effluent for the duration of the modulation time. These requirements significantly constrain physical method parameters, such as the column dimensions (combination of internal diameter, length, and particle size), ^1^D and ^2^D flow rates, and the volume of the sampling loops.

In the following paragraphs, we describe guidelines to help separation scientists understand the rationale for selecting optimal physical parameters in an LC × LC method. For in‐depth treatments, the reader is referred to systematic studies 31, 34, 35.

#### 2.3.1 How to choose the column dimensions for an LC × LC method

Finding the appropriate column dimensions requires understanding of several theoretical aspects. Schoenmakers et al. developed a protocol for the design of an efficient LC × LC separation system 36. According to this protocol, the maximum (^1^D) analysis time and pressure drop should first be defined by the analyst. The maximum analysis time is typically rather long, i.e., several hours when using an HPLC system (40 MPa pressure limit) or 30–100 min when using contemporary UHPLC instrumentation (100–150 MPa).

In LC × LC, a slow ^1^D separation (typically using a shallow gradient) is sampled multiple (often more than 100) times by a fast ^2^D separation. The selection of the columns requires several factors to be considered (a discussion of the advantages and disadvantages of different column combinations is reported in Section 3 of this review). Ideally, the ^1^D separation should feature the highest separation power for the sample mixture, whereas the ^2^D should be fast, efficient, and compatible with the detector chosen.

To allow the ^1^D separation to be suitably slow, the column is relatively long (100–250 mm) which favors high efficiencies. In contrast, the ^2^D column is typically short (50 mm or less) to ensure fast separations. The length of the ^2^D column is particularly important when gradient separations are used. Short columns allow gradients with high volume ratios (*t_G_*/*t*_0_ = duration of gradient/column dead time), while maintaining short cycle times and reducing the time needed to equilibrate a column after a gradient.

**Figure 6:**
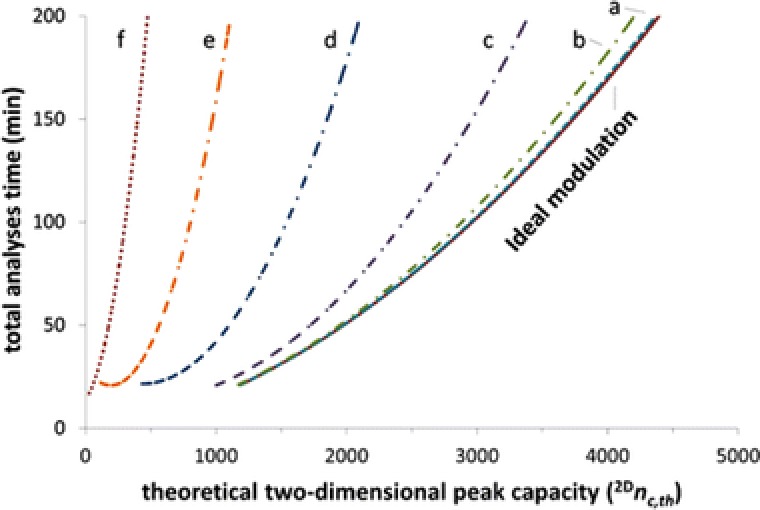
Pareto‐optimal fronts of theoretical peak capacity versus total analysis time column diameter ratios (^2^ *d*_c_/^1^ *d*_c_ *)* of (a) 7 (partly obscured by the drawn line representing ideal modulation), (b) 4, (c) 2, (d) 1, (e) 0.5, and (f) 0.25. Reprinted with permission from 56. Copyright 2017, American Chemical Society

The choice for an appropriate combination of column diameters is tightly connected to the injection band broadening in the ^2^D separation and the dilution factor (ratio of the analyte concentration in the injected sample and at the entrance to the detector). The influence of the ^1^D and ^2^D column diameters (^2^ *d*_c_/^1^ *d*_c_ *)* on the theoretical peak capacity and the analysis time is illustrated in Fig. 6.

The pareto‐optimality results show that the highest peak‐production rate (*n*_c_/*t*_anal_) is achieved with a diameter ratio (^2^ *d*_c_/^1^ *d*_c_) between 7 and 4. An example of a good choice 1 mm ID ^1^D column in the ^1^D in combination with a 4.6 mm id ^2^D column. The rationale of this choice is that 1 mm ^1^D column allows separations at low volumetric flow rates (e.g., 10 μL/min), while maintaining reasonable linear velocities (e.g., 0.21 mm/s). This results in small volume per collected fraction, reducing the ^2^D injection volumes. Similarly, having a wider ^2^D column (e.g., 4.6 mm) increases the volume of the column used, so that larger volumes can be injected without significantly affecting the separation performance. Furthermore, wider ^2^D columns can be operated at higher flow rates, reducing the delay of gradient delivery caused by system dwell volumes.

**Figure 7:**
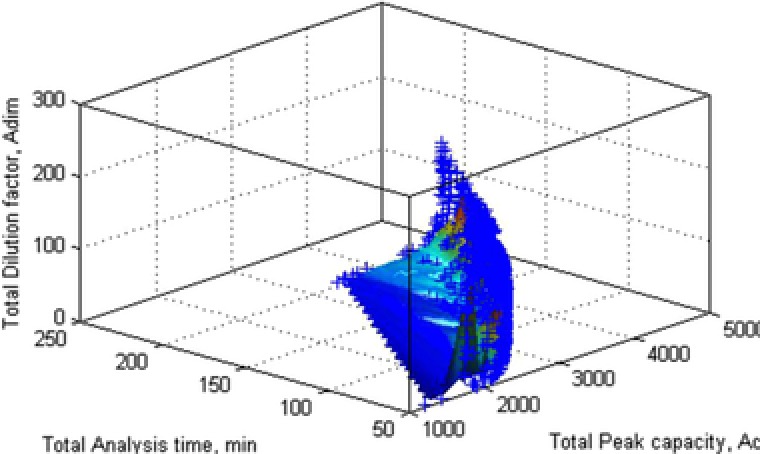
Pareto‐optimal surface resulting from optimizing total peak capacity, total analysis time, and total dilution for an LC × LC system at a given maximum pressure. Reprinted with permission from 31. Copyright 2017, American Chemical Society

However, the combination of (i) a high ^2^ *d*_c_/^1^ *d*_c_ ratio, (ii) high ^2^D flow rates, and (iii) sampling peaks multiple times lead to dilution of the analytes in large amounts of solvent. This can be taken into account by introducing a dilution factor 31, 36. As can be seen from Fig. 7, an analytical scientist must strike a compromise between analysis time, peak capacity, and dilution factor.

Another important physical parameter in designing an LC × LC system is the choice of the stationary phase morphology used in each dimension. Currently the majority of LC × LC methods are developed using silica based fully‐porous particles, functionalized with chemistries that depend on the separation modes used. Other types of technologies, such as monoliths (organic or silica based), core–shell particles, and polymeric beads may be used in specific applications. In an LC × LC method, the ^2^D separation column is subjected to severe stress, with fast gradients delivered at high linear flow velocities, often at elevated temperatures (e.g., >50°C) in multiple injection cycles (with accompanying pressure pulses) for each analysis. Therefore, robustness is an essential parameter in considering the ^2^D column in an LC × LC system.

Sub‐2 μm particles and UHPLC system technology are now commonly used in both separation dimensions. For the ^2^D separation the main advantage arises from the high efficiency per unit time and from the reduced influence of high linear velocities. Relatively long ^1^D columns can be used to maximize the separation performance of LC × LC systems 37. Moreover, UHPLC systems exhibit reduced dispersion, resulting in less extra‐column band broadening and shorter dwell times 31.

### 2.4 Practical considerations

Many additional parameters should be taken into account. For example, temperature and mobile‐phase composition (viscosity) will affect both the chemical (selectivity) and physical (efficiency) optimization, so that these two processes cannot strictly be separated. In the opinion of the authors, the impact of each of this parameter should be considered, but during the design and development of an LC × LC method pragmatic choices must be made to establish whether the selected separation principles may succeed in answering the analytical question. Typical initial conditions for testing an analytical‐scale LC × LC setup may include a 1 mm (or 2.1 mm) id column of 150 mm length in the ^1^D with 3–5 μm particles, run at 10–100 μL/min, using a shallow gradient (between 60 and 200 min). The loop size for collecting fractions should have a volume between 20 and 60 μL. The ^2^D should be optimized to cycle times (analysis and column equilibration) between 20 and 120 s. If at all possible, initial testing should be performed using a reference sample or a representative standard mixture, so that the process (and the analyst) is not frustrated by running out of sample. In that case, relatively high ^2^D flow rates can initially be used. When solvent consumption is not an initial concern, a 50 mm × 4.6 mm id UHPLC ^2^D column packed with sub‐2 μm particles can be operated at flow rates up to 4 mL/min (or higher). When proper separation conditions are selected, such a setup should offer high peak capacities. When the availability of sample is an issue from the start or when a detector with low flow limits (e.g., split‐less coupling to MS) is targeted, we suggest to start testing separation methods with a 50 mm × 2.1 mm id UHPLC ^2^D columns run between 0.5 and 0.7 mL/min. With such a setup, part of the potential peak capacity will be sacrificed to gain sensitivity and reduce solvent consumption.

**Figure 8:**
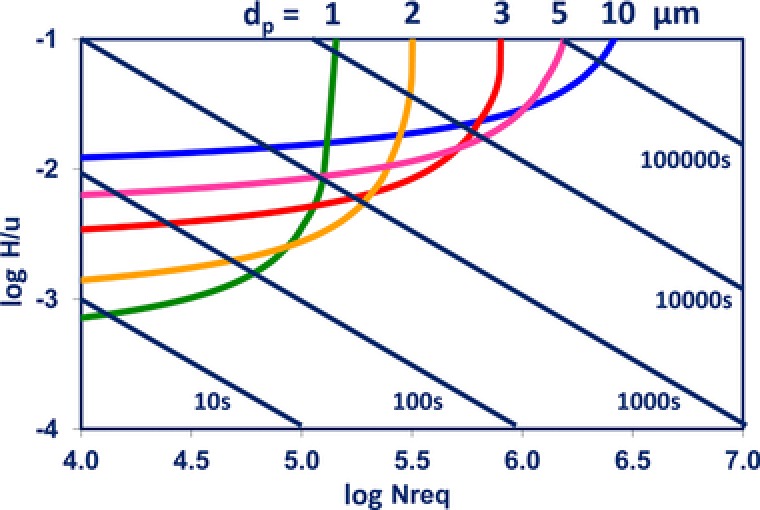
Example of a Poppe plot. Δ*P* = 40 MPa, viscosity 10^–3^ Pa·s, Knox reduced plate‐height equation *h* = 0.5*ν* ^1/3^ + 3/*ν* + 0.1*ν*.

To establish optimal particle sizes in each dimension, the above parameters can be combined with the estimated mobile phase viscosity and temperature to establish Poppe plots 38, 39 or, more generally, kinetic plots 40, 41. In a Poppe plot, the time required per theoretical plate is plotted against the total number of theoretical plates. An example for typical LC conditions is shown in Fig. 8. Each curve represents a distinct particle size and the diagonal lines depict discrete holdup times. Somewhat counterintuitively, difficult separations requiring high efficiencies (*N* ≥ 100 000) and long analysis times in the ^1^D (*t*_0_ above, say, 10 min or analysis time above 100 min) are seen to benefit from relatively large particle diameters (≥5 μm). Contemporary sub‐2 μm particles are more favorable for relatively simple (*N* ≤ 25,000), fast (*t*_0_ ≤ 1 min) separations. In all cases, efficient separations achieved in the ^1^D may be jeopardized by undersampling. Approximately, the time allowed for ^2^D separations is ½ √^1^ *N* times shorter than the holdup time in the ^1^D (^1^ *t*_0_) 36, as this allows two cuts across the fastest peaks (*t_R _*≈ *t*_0_). In practice, bearing in mind that gradient elution is predominantly used in the ^1^D and that relevant peaks ideally do not elute around *t*_0_, a factor of about 0.15 √^1^ *N* suffices. Thus, if ^1^ *N* = 10 000 and ^1^ *t*_0 _= 5 min, the ideal modulation time is of the order or 20 s. Because it is impossible to choose “ideal” values for all parameters in LC × LC, optimization amounts to finding the most attractive compromise between all possible settings.

#### 2.4.1 Modulator‐loop size

The volume of the modulation loops determines the volume of ^1^D effluent that can be stored and the ^2^D injection volume. In general, to limit injection band‐broadening effects, the volume injected should not exceed 15% of the column dead volume 42. However, depending on the type of LC used (retention mechanism, elution mode) and the composition of the injection solvent, the maximum permissible volume can be <1% (e.g., injection of fully aqueous sample in HILIC) or >50% (e.g., injection of sample dissolved in water before a gradient IEC separation 43).

**Figure 9:**
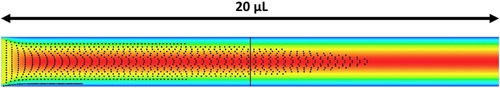
Visualization of the situation after a 20 μL loop has been filled for 1 min at 10 μL/min. The ^1^D eluent species are depicted as dots. The magnitude of the flow velocities is represented by the colour. Analyte molecules to the right of the vertical line in the middle would be lost if the loop volume and sample volume were equal. Visualization created using computational fluid dynamics by Dr. Suhas Nawada (University of Amsterdam)

One often overlooked aspect is the effect of the parabolic flow profile. The LC pumps apply pressure to push the mobile phase through narrow channels (e.g., the sampling loop, tubing, and the interstitial channels in the column), friction near the walls results in a parabolic flow profile, also known as Hagen–Poiseuille flow. The importance of this flow profile becomes clear from Fig. 9 which considers the filling of a sample loop of 20 μL at a flow rate of 10 μL/min for the duration of 1 min. In an unperturbed, laminar system the flow profile is parabolic, the velocity in the center is twice the average velocity. Consequently, when filling a 10 μL loop with 10 μL of sample, analyte molecules migrating predominantly along the central streamlines may be lost. In the absence of diffusion, the volume of the loop should be double the volume of the collected fraction. Although this effect is mitigated by axial diffusion, it is advised to use sampling loops with twice the modulation volume to avoid loss of ^1^D effluent.

## 3 SELECTION AND COMBINATION OF SELECTIVITIES

### 3.1 Selection of selectivities

**Figure 10:**
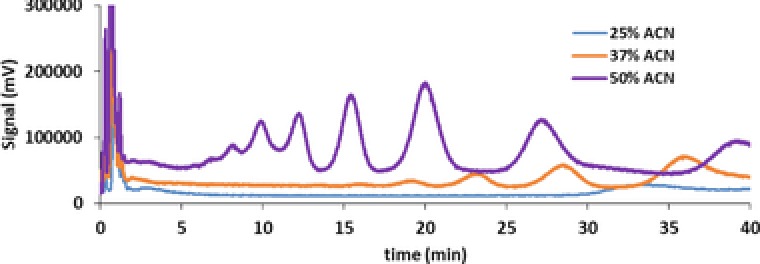
Overlay of chromatograms displaying the separation of an industrial mixture of anionic surfactants with the mobile phase comprising 25% (blue), 37.5% (orange), and 50% (purple) acetonitrile (see Supporting Information Section S3 for the analysis method details used to record this data)

After treating the fundamental concepts of LC × LC method development, we move toward the selection of two candidate selectivities for the separation of the analyte mixture. In this context, selectivity represents the extent to which the structural elements of the analyte molecules influence the retention behavior. Hence, a decision on the separation selectivities requires an assessment of the most‐important structural molecular descriptors for the analyte mixture (if known), described by Giddings as the “sample dimensions” 18, 44. Once key molecular descriptors are identified, the analyst can choose suitable retention mechanisms through selection of appropriate stationary phases (i.e., columns) and tailor the selectivity in both dimensions by tuning the mobile phase composition (and its variation in time). The toolbox of the LC analyst offers a wide array of retention mechanisms that can be divided into a number of classes as shown in Table 1. Typically, the stationary phase is selected first in conjunction with the retention mechanism. The mobile phase can accentuate, moderate, convolute, or even nullify the retention mechanism associated with the stationary phase. For example, when using acetonitrile 45 or tetrahydrofuran 46 in conjunction with a phenyl‐modified stationary phase the π–π interactions of aromatic analytes are inhibited, thus weakening or even negating the specific interaction 45. Potential convolution of the intended retention mechanism is sometimes encountered in IEC. IEC stationary phases may feature hydrophobic interactions retention increments 47, 48. Mixed mechanisms are sometimes deliberately exploited. Pure IEC would require adjustment of the mobile phase to counteract other interactions 49. An example is shown in Fig. 10 (see Supporting Information Section S3 for the analysis method details used to record this data).

**Table 1:** Overview of retention mechanisms

	Mechanism	Acronym	Selectivity	Common stationary phase (SP) selectors
1	Reversed phase	RP	Hydrophobicity, Chain length, carbon skeleton	Alkyl (hydrocarbon: C1 to C30; most commonly C18), cyano (π–π)*, phenyl (π–π)*, carbon‐clad zirconia (or graphitized carbon), PEG.
1	Ion pairing	IP	Hydrophobicity, suppression of analyte ionization (acid/ bases)	Alkyl (hydrocarbon)
1	Hydrophobic interaction	HIC	Hydrophobicity	Short‐chain alkyl hydrocarbons (C4 to C8)
2	Normal phase	NP	Polarity, Functional groups	Bare silica, Amino‐propyl, diol, cyano
2	Argentation	AgLC	Degree of saturation, *cis‐trans* isomers	IEC columns (e.g., sulfonic acid) or bare silica loaded with silver ions
2	Hydrophilic interaction	HILIC	Hydrophilicity, polar character	Zwitterionic: sulfobetain, phosphocoline; Basic: amino propyl; and Neutral: diol, amide
3	Ion exchange	IEX	Charge, ionic interactions	SCX: sulfonic acid; WCX: carboxylic acid; WAX: triethyl amine; and SAX: quaternary Amine
4	Size exclusion	SEC	Molecular size, Molecular weight	Crosslinked poly(styrene – divinyl‐benzene) or methacrylate porous beads (SEC organic solvents); Polar‐functionalized porous silica (SEC aqueous)
5	Mixed mode	MM	Combination of retention mechanisms	Anion‐exchange/RP (AEX/RP), Cation exchange/RP (CEX/RP), AEX/CEX/RP; AEX/HILIC, CEX/HILIC, AEX/CEX/HILIC
6	Chiral	Chiral	Selector‐specific chirality	Variety of selector depending on the application. Most common are based on polysaccharide derivatives (chiral carbamate/ benzoate polymers of cellulose and amylose)
7	Affinity	Affinity	Selector‐specific affinity	Stationary phases with chemically bonded antigens or proteins

The retention mechanism can also be nullified by the mobile phase. One example is the use of C18 columns for SEC analysis of polymers. Usually, such columns are used with aqueous‐organic solvents as mobile phase, often with a gradient toward a less‐polar solvent, such as acetonitrile or methanol. However, the same column can be used in conjunction with tetrahydrofuran as mobile phase, such that all hydrophobic interactions are negated and the column essentially functions as a SEC column. Clearly, the selection of the mobile phase is just as decisive as selection of the stationary phase.

### 3.2 Compatibility of mobile phases

The order in which two separation dimensions are combined is of utmost importance, related to the compatibility of the mobile phases. This is one of the most difficult challenges in developing LC × LC separations. Fractionation of the ^1^D effluent and subsequent injection into the ^2^D presents the sole physical connection between the two dimensions. It is crucial that the ^1^D mobile phase is compatible with the ^2^D separation method, or that potential detrimental effects on the ^2^D separation are known, so that appropriate measures can be taken, if possible. Aside from detrimental influences on the ^2^D selectivity, the ^1^D eluent may also have a devastating effect on the ^2^D separation in general. One potential problem is flow instability during the transfer and injection of a fraction from ^1^D to ^2^D. If the viscosity of the ^1^D fraction is relatively high compared to that of the ^2^D mobile phase, injection into the ^2^D may result in viscous fingering 50. The effect occurs at the interface between the two liquids, as the low‐viscosity ^2^D mobile phase potentially penetrates the high‐viscosity sample plug during percolation through the porous media, resembling fingers of mobile phase. The chromatographic performance is jeopardized and in severe cases peak splitting may be observed 51.

Another problem may arise from the difference in solvent strength of the sampled ^1^D fraction and the ^2^D mobile phase, particularly if the eluent strength of the ^1^D effluent is too strong for retention to occur in the ^2^D column. In this case, analytes in the fractionated plug will be dragged through (part of) the ^2^D column in the sample plug. As the plug migrates through the column, analytes will perpetually disperse in both directions. An extreme case may be observed for large molecules, when size‐exclusion conditions prevail in the solvent plug. Because large molecules move faster than the solvent front, they will move faster than the mobile phase and focus at the front of the solvent plug. In front of this plug, the mobile phase is relatively weak. Analytes will slow down and be caught up again by the plug. Only analytes at the rear of the plug, where size‐exclusion conditions do not prevail, will be retained and adsorption effects become prevalent. The result is the so‐called breakthrough phenomenon 52, with two peaks appearing for the affected analytes. Typically, the breakthrough peak will elute with the dead volume (*t*_0_), whereas the—typically much smaller—“real” peak will elute at its normal location. Notorious combinations which are known to give rise to breakthrough distortions are SEC (organic) × RPLC and, to a lesser extent, NPLC × RPLC. In general, a decrease of injection volume and/or strength of the injection solvent, may significantly decrease the occurrence of these phenomena 52.

**Figure 11:**
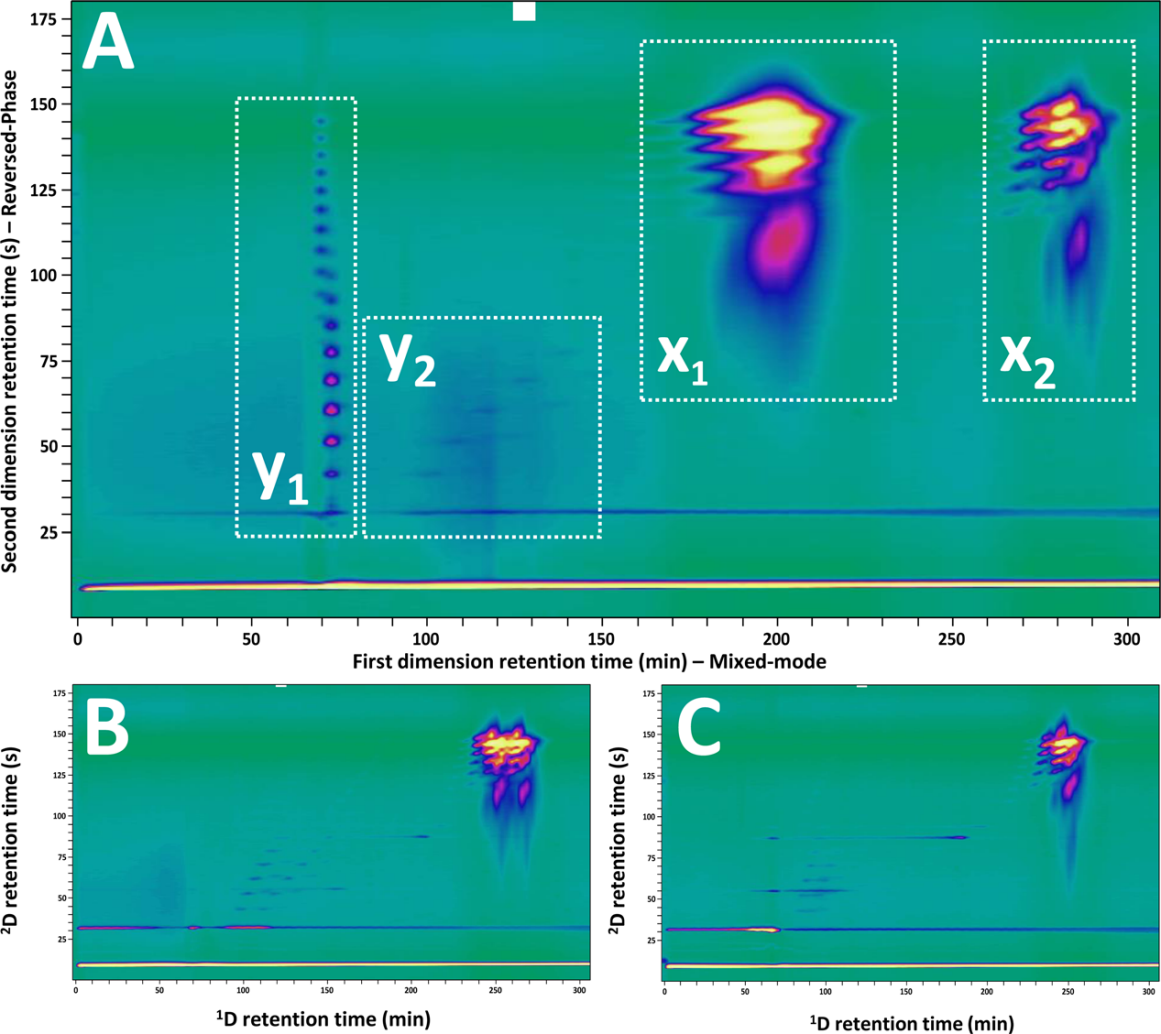
Example of detrimental effects of incompatibility of ^1^D injection solvent and mobile phase. Mixed mode × RPLC separation of industrial surfactants 22. Injection volume (A) 20 μL, (B) 10 μL, and (C) 5 μL

An example of the extent of the detrimental effects of solvent incompatibility is shown in the mixed mode × RPLC separation of industrial surfactants (Fig. 11A). The injection solvent (2‐propanol) is highly viscous and a strong eluent. With an injection volume of 20 μL, the solvent plug does not dissolve rapidly in the column and several groups of peaks appear in the chromatogram. Mass spectra indicated the composition of distributions X1 and X2 to be similar. This was likewise found for Y1 and Y2. Species that eluted in distribution Y1, were also found to elute at distribution Y2. Decreasing the injection volume to 10 μL (Fig. 11B) and 5 μL (Fig. 11C), significantly reduced the extent of the detrimental effects. While it is difficult to precisely determine the contributions of each of the phenomena discussed in this section, the misleading separation shown in Fig. 11A underlines the importance of matching the injection solvent with the mobile‐phase solvent system, both in terms of solvent strength and viscosity.

It should be noted that advantageous and disadvantageous injection‐solvent effects are also encountered in 1D LC. The most favorable injection solvents (in 1D‐LC) and transfer solvents (in LC × LC) are weaker eluents than the pertaining mobile phase.

### 3.3 Solutions to circumvent solvent incompatibility

Problems arising from the solvent strength or viscosity mismatch (or a combination of the two) of the fraction transferred from the ^1^D can lead to severe peak distortion and losses of separation power in the ^2^D. To circumvent this, different research groups have proposed alternatives to the conventional loop‐based fractionation interface. We refer to this conventional approach as “passive modulation”. In contrast, all approaches in which the ^1^D effluent is modified before ^2^D injection are referred to as “active modulation”. To reduce the deterioration of RPLC × RPLC performance due to injection‐solvent effects (e.g., solvent strength or pH), Stoll et al. have demonstrated the feasibility of adding a postcolumn dilution solvent before 53, 54 and recently within the modulation interface (“active solvent modulation” 55). Weakening the ^1^D effluent increases the retention on the ^2^D column head (focusing effect) and this improves peak‐shape symmetry, and enhances peak height and peak capacity, despite the larger volume injected.

It has been shown that it is possible to substitute the empty loops of the modulation interface with trap columns. This approach can be used for column combinations such as SEC × RPLC, SCX × RPLC, RPLC × RPLC, and HILIC × RPLC. It allows reduction of the ^2^D injection volume and manipulation of the solvent (e.g., desalting of the fractions from the ^1^D 56). Using this stationary‐phase assisted modulation approach we could increase the column diameter of the ^1^D with respect to the ^2^D (^1^ *d*_c_ *__*> ^2^ *d*_c_), greatly reducing dilution in LC × LC, while reducing the overall analysis time 56, 57.

The coupling of NPLC and RPLC causes severe injection problems, deriving from the solvent‐strength and miscibility incompatibility of the two solvent systems. For this column combination, researchers have been designing modulation interfaces that allow for solvent exchange or enable a significant reduction of the volume injected. These concepts include thermal modulation (in‐column focusing 58) and vacuum‐evaporation modulation 59, 60. Recently Li et al. succeeded in coupling NPLC × RPLC online using a modulation interface with ^1^D trap columns, dilution flow, evaporation, and ^2^D trap columns (“thermal evaporation assisted adsorption interface” 61), allowing for complete removal of the NP solvent.

These approaches have, to different extents, proven effective at reducing incompatibility problems, but to the date they have not reached a level of maturity that would allow their use in routine LC × LC applications. Currently, issues connected with solvent‐strength compatibility can be resolved with relatively easy modifications of commercially instrumentation, such as dilution of the ^1^D eluent or solid‐phase assisted modulation. Issues involving solvent‐strength and miscibility, like those encountered in coupling NPLC and RPLC, remain a challenge for which to this moment only the introduction of very small volumes to the ^2^D 62 provides an ad hoc solution.

### 3.4 Combining selectivities

When developing an LC × LC method, the choice between combinations of stationary‐phase chemistry and mobile‐phase composition is large. Each combination potentially benefits or suffers from various factors. Moreover, only a limited number of chromatographic mechanisms have been used in LC × LC and reported in literature, although for certain applications it would be interesting to explore other combinations.

To guide the reader, we provide a comprehensive overview of strengths and weaknesses of possible combinations in Tables 2 and 3 below, summarizing obstacles and opportunities arising from the combination of the most common separation mechanisms. Advantageous and detrimental factors are depicted using symbols for each combination including the (lack of) orthogonality, potential peak capacity, duration of column re‐equilibration, and solvent compatibility. We emphasize that the symbols should be interpreted as our advice to consider for a specific combination. We do not want to discourage the pursuit of seemingly unfavorable combinations. In fact, the references provided for a number of combinations showcase how smart method development can alleviate many of the pitfalls. See Supporting Information Section S4 for a version of Table 3 with references.

**Table 2:** Overview of symbol clarifications as used in Table 3

**Symbol**	**Meaning**	**Used For**	**Description**
A	Adsorption	^2^D	Lengthening of elution time due to injection solvent. Applies exclusively to SEC.
B	Breakthrough/Peak distortion	LC × LC	Anomalous early elution of analytes injected from ^1^D to ^2^D. See section 3.2 for more information.
E	Easy to modulate	LC × LC	Ease of developing active modulation methods (e.g., trap columns or solvent admixing).
F	Fast separation	^2^D	Method with short analysis times (e.g., <1 min)
H	High‐resolution separation	^1^D, ^2^D	Method capable of high peak capacity.
I	Isocratic	^1^D, ^2^D	Possibility of (easily) running isocratic methods, reducing the complexity of the setup.
M	MS compatible	^2^D	Possibility of using volatile mobile‐phase additives and achieving good MS sensitivity.
O	Orthogonal	LC × LC	Degree of independence of two separation mechanisms, assuming that the analyte mixture exhibits sample dimensions targeted by the two dimensions.
P	Applicability	LC × LC	Usefulness of the resulting separation.
Q	Column reequilibration	^2^D	Speed of column reequilibration.
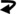	Reversed‐order recommended	LC × LC	Recommended to consider the reversed order of the mechanisms.
S	Selectivity/Specificity	^1^D, ^2^D	Capability of the separation method to separate based on chemical characteristics of sample components (e.g., shape, orientation, composition/ sequence)
X	Solvent compatibility	LC × LC	Extent of (in)compatibility of ^1^D effluent and ^2^D eluent.
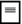	Reference available in text		Example of references can be found in Supporting Information Section S2.
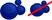	Polymers		Suitable/unsuitable for separations of polymers.
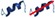	Proteins		Suitable/unsuitable for separations of proteins.

**Table 3:** Overview of the possible online LC × LC combination using the most‐common forms of LC separations

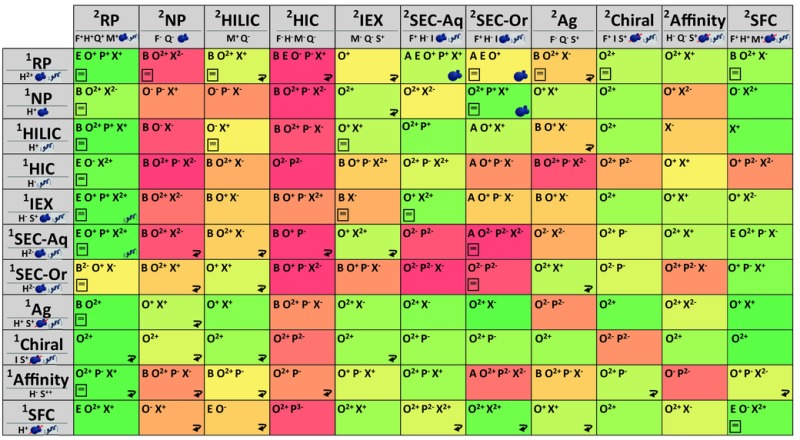

In the following sections, the feasibility of each combination of generic retention mechanisms plus a selection of interesting mechanisms is discussed. Where applicable, we refer the reader to useful applications and reviews which offer a great deal additional information 63. Applications in life sciences 64, 65, food 66, 67, 68, 69, polymers 70, 71, 72, and traditional Chinese medicine 73 are covered by recent reviews.

#### 3.4.1 RPLC

In RPLC, separation is achieved based on differences in hydrophobicity of the analytes. Typically, columns packed with alkyl‐modified silica particles are used, enabling hydrophobic interaction with the hydrocarbon sections of the analyte. Specific structural features of analyte molecules may be targeted when using packing materials with additional functional groups (e.g., cyano stationary phases).

Its versatility and applicability make RPLC the most popular retention mechanism in LC × LC. As is also reflected in Table 3, RPLC is especially popular as ^2^D separation, because it adds fast, complementary, and nonspecific interaction‐based separation to a specific ^1^D mechanism. Moreover, RPLC may provide high efficiencies in fast, high‐pressure separations using sub‐2 μm particles 74. Elevated temperatures can be used to further improve the separation performances 75, 76. Furthermore, partial equilibration using conditioning volumes down to a single column volume has been shown to provide reliable, repeatable separations in gradient elution 21.

Gradient elution is predominately used for RPLC separations to accommodate a broad range of analytes. A particularly attractive property that explains the use of RPLC ^2^D separations is their general compatibility with MS, provided that no ion pair is used.

One potential challenge is the compatibility with the ^1^D mobile phase. Combinations such as NPLC × RPLC and SEC (organic) × RPLC are notorious for resulting in detrimental solvent incompatibility effects (see Section 3.2). RPLC separations can also be employed in the ^1^D if the opposite combination is not compatible.

The flexibility of RPLC has also prompted chromatographers to combine two RP separations in the form of RPLC × RPLC. While such a combination might initially seem less favorable in terms of orthogonality, it is good to remember that the selectivity of RP can be tuned quite strongly in various ways (Table 1). A nonspecific RPLC separation can be combined with a more‐specific RPLC selectivity. A good example is the use of a carbon‐clad zirconia column as ^2^D, which has shown to provide significantly different selectivity 77, 78. PEG stationary phases have also shown to provide unique selectivities relative to C18 79, whereas pentafluorophenyl‐propyl‐silica was found to be similar in selectivity to C18 80. However, the selectivity between two RPLC separations can also be tuned using more‐subtle effects. End capping prevents hydrogen bonding between hydrogen‐acceptor moieties and remaining free silanol groups to safeguard orthogonality 81. The group of Snyder published an excellent series of articles on column selectivity in RPLC, addressing the various selective chemistries for use in RPLC 46, 81, 82, 83, 84, 85, 86, 87.

Selectivity may also be modified by changing the pH or adding an ion‐pairing agent in one of the two dimensions. An example of the first is the separation of the antibody–drug conjugate ado‐trastuzumab emtansine by LC × LC–MS 88. The method developed by Sandra et al. combined two RPLC separations, one at high pH and the other at low pH, with shifting gradient assemblies to obtain good separation.

The seemingly endless number of RPLC selectivities have prompted researchers to develop tools to aid in characterizing and selecting appropriate options. The Abraham model utilizes solvation equation based on linear‐free‐energy relationships and can be used to compare stationary phases and to classify the selective interactions between analyte and stationary phase. The hydrophobic subtraction model allows improved understanding of the polar interactions by removing the hydrophobic contribution to the retention 89, 90. The hydrophobic subtraction model has been used to identify orthogonal RPLC dimensions in LC × LC 80.

Despite the large array of options to manipulate RPLC selectivity in both dimensions, a degree of correlation is intrinsically unavoidable. It is therefore not surprising that shifting gradients have been applied in combination with different stationary‐phase selectivities to improve the separation 24, 91.

#### 3.4.2 Normal‐phase LC

Contrary to RPLC, NPLC utilizes polar sorbents as stationary phases to separate sample components based on polar moieties. There is a broad choice of solvents, with the least polar eluent being most retentive. The polar solvent component is preferentially adsorbed on the column. In the extreme case in which the polar component is water in very low concentrations (typically in acetonitrile) an aqueous solvation layer is formed on the polar stationary phase we speak of HILIC.

When using NPLC in LC × LC solvent‐incompatibility caveats are challenging. For example, in RPLC × NPLC the aqueous ^1^D fractions significantly hinder successful modulation. For some quite nonpolar samples this problem may be avoided by using RP in non‐aqueous mode (NARP). Alternatively, the analyst may opt to dilute the aqueous ^1^D fraction with acetonitrile to make it compatible with ^2^D HILIC. An exception to the poor compatibility of NPLC is its combination with organic SEC. NPLC × SEC has been widely applied to the analysis of polymers with the NP separation often carried out at (isocratic) critical 92, 93 or (gradient) preudo‐critical 94 conditions, where retention is independent of analyte molecular weight 95, 96, 97.

The solvent compatibility issues are less dominant when NPLC is used as ^1^D separation. This choice is aided by the relatively poor analysis and re‐equilibration times when using ^2^D NPLC in gradient mode. NPLC × RPLC was reported for the separation of cold‐pressed lemon oil 62, alcohol ethoxylates 98, and oligomers 99.

Active modulation techniques (e.g., stationary‐phase assisted modulation), which may be used to overcome compatibility problems are difficult to apply because the ^1^D effluent is a strong solvent on RP traps. Efforts to improve compatibility thus focus on the removal or replacing of the strong solvent fraction of the ^1^D effluent. An example is the evaporation approach 100, which was recently applied for the analysis of toad skin 61.

##### 3.4.2.1 Argentation (silver‐ion) normal‐phase chromatography

Argentation (silver‐ion) chromatography (AgLC) is a form of normal‐phase chromatography, where a silica packing material is treated with an aqueous solution of silver nitrate. Using an organic mobile phase with a small fraction of polar solvent, selective retention is obtained through π–π interactions between the double bonds in unsaturated analytes and the silver ions. In essence, the separation is based on differences in the extent and location(s) of unsaturation and its main application is to the analysis of lipids. Similar to NPLC, AgLC has been applied mainly as ^1^D separation for the AgLC × RP analysis of rice oil 101, soybean oil 102, peanut oil, and mouse tissue 103.

#### 3.4.3 HILIC

Introduced by Alpert 104, HILIC conditions allow separation based on hydrophilicity. A variety of stationary phase sorbents can be used to tailor the HILIC retention mechanism to the sample by improving specific retention for specific analytes. For example, the use of zwitterionic moieties in HILIC packings give rise to additional ionic interactions, creating a contribution of analyte charge to retention, as in IEC. Alternatively, ion‐pairing agents may be added to the mobile phase similar to ion‐pair chromatography for the separation of charged and ionizable compounds. Although becoming increasingly popular, the exact retention mechanism of HILIC is still not well understood and researchers have been working on modeling the retention behavior in HILIC for various stationary‐phase sorbents to aid method development and gain better understanding of the interactions 105, 106, 107. The latest development in this research area have recently been reviewed 108, 109.

Application of HILIC in LC × LC is, however, largely limited to use in the ^1^D. This is related to the lengthy re‐equilibration of HILIC separations, due to the slow desorption and reformation of the aqueous layer on the stationary phase after each injection 110. D'Attoma and Heinisch applied RPLC × HILIC for the separation of a tryptic digest of three proteins and compared the results with an RPLC × RPLC separation. The authors concluded that the RPLC × HILIC method did not suffer from peak‐shape distortion as a result of overloading, but did suffer from injection effects 111. Holčapek et al. applied RPLC × HILIC–MS for the analysis of complex lipidomic samples 112. Because the authors focused on phospholipids, the gradient span in the HILIC dimension could be narrow, allowing relatively short re‐equilibration times. Typical applications of HILIC in LC × LC use a RPLC ^2^D separation. Examples include separations of cocoa procyanidins 113, anthocyanins in red wine 114, phosphatidylcholine isomers 115, and surfactants 57. The latter example involved the use of active modulation to significantly reduce the analysis time and dilution of the HILIC × RPLC separation.

Similar to RPLC separations, different specific interactions can be targeted by different HILIC columns (e.g., acidic, basic, zwitterionic, amide, diol) in each dimension to establish HILIC × HILIC. Wang et al. developed an online HILIC × HILIC system and applied it to separate saponins from *Quillaja saponaria* 116. While there was still room for improvement in terms of chromatographic efficiency, mainly in the ^2^D, the authors did demonstrate the potential of the system in terms of orthogonality.

#### 3.4.4 Ion‐exchange chromatography

For separation purely on charge properties, several modes of IEC exist, depending on the stationary and mobile phases. In strong IEX, a permanently charged sorbent (e.g., quaternary ammonium for anion‐exchange, SAX, or sulfopropyl for cation‐exchange, SCX) is used for the separation of oppositely charged analytes and retention is reduced by the (gradual) increase of a salt‐buffer concentration in the mobile phase. In weak ion‐exchange, elution of retained analytes may also be influenced by altering the pH to (de)protonate the IEX sorbent (e.g., diethylaminoethyl for anion‐exchange, WAX, or carboxymethyl for cation‐exchange, WCX) or the analyte itself.

Typically, IEX is employed in the ^1^D due to (i) the long re‐equilibration times for IEX gradients and (ii) the incompatibility of the salt buffers used with popular detectors such as MS and ELSD. IEX combined with RP is a common approach for the analysis of biomolecules, such as proteins. Perhaps one of the most familiar examples is the multidimensional protein‐identification technology (MudPIT) 117, which uses stop‐flow modulation to combine SCX with RPLC. Vanhoenacker compared the separation of monoclonal antibody digests by SAX × RPLC with RPLC × RPLC 118. Stoll also studied antibody separations using SCX × RPLC–MS and selective‐comprehensive SCX × RPLC–MS 119.

One issue that may jeopardize the orthogonality of an IEC × RPLC system is the influence of the charged analyte moieties on the hydrophobic interactions in RPLC. If the charge of the analyte is its main sample dimension targeted by the ^1^D separation, then it should preferentially not affect the ^2^D separation. To achieve this, ion‐pairing agents have been used in the RPLC separation. An example is the SAX × RPLC system for the separation of synthetic dyes 49. The authors reported, however, that ion‐pairing agents with large hydrophobic groups could increase hydrophobic retention for analytes with increasing numbers of charged moieties, signifying that the ion‐pairing reagent must be selected with care. Vonk et al. used active modulation to improve the high‐resolution‐MS sensitivity for a SCX × RPLC–MS system for analyzing the proteome of *Saccharomyces cerevisae* 56.

Separation systems which apply IEC in ^1^D with other retention mechanisms, such as NPLC, HILIC, HIC, and AgLC in ^2^D, may suffer from peak distortion and breakthrough phenomena. Adsorption effects may occur in ^2^D SEC organic (Table 3).

In IEX the key separation dimension is (the number of) charged moieties of the analytes. Thus, it may appear strange to pursue comprehensive 2D IEX (IEX × IEX). However, Shellie et al. 120 combined a cation‐exchange separation with an anion‐exchange separation. They obtained a good selectivity combination, essentially making use of the mixed‐mode interactions that these columns presented.

#### 3.4.5 Size‐exclusion chromatography and hydrodynamic chromatography

In SEC, separation is based on the molecular size of the analyte molecules in solution. Large molecules are excluded from the pores and will travel faster through the chromatographic column in comparison with molecules that can (partially) permeate the pores. Interaction between the analytes and the stationary phase is avoided by using mobile phase solvents that interact more strongly with the analytes than the stationary‐phase surface. In the case of charged polymers, buffers are added to inhibit electrostatic interactions. The resolution obtained in SEC is limited, but the technique is widely used to obtain the molecular‐weight distribution of the analyte mixture. Large analytes that are excluded of the pores may be separated based on wall exclusion, through hydrodynamic chromatography (HDC) 121.

A special form of polymer chromatography is “critical chromatography” or LC at the critical conditions (LCCC) 92, 93, 94, 122. In LCCC, the mobile phase is chosen such that retention is independent of the molecular weight of the analyte polymers. LCCC can be seen as a special isocratic form of NPLC or RPLC and is not treated as a distinct mechanism in Table 3.

From a classical perspective, SEC benefits from large columns with large pore volumes 123 and thus is easier to use in the ^1^D in terms of achieving the highest possible resolution 124. However, depending on the ^2^D separation mechanism, breakthrough and other solvent incompatibility effects may occur. In case of aqueous SEC as ^1^D with a ^2^D RPLC separation, peak focusing is straightforward 125. On the other hand, ^2^D SEC potentially suffers from limited resolution. However, recent studies have shown the potential of fast SEC, using UHPLC technology 126, 127. More recently, the use of core–shell particles in ^2^D SEC to improve resolution was demonstrated 128 and this was later confirmed 129. SEC is an intrinsically isocratic separation. When used as ^2^D separation, there is a wider choice of detectors and re‐equilibration is not necessary. In fact, overlapping injections allow cycle times much shorter than the ^2^D analysis time 130.

It is thus not surprising that LC × SEC has been widely applied for the separation of synthetic 97, 131 and (modified) natural 132 polymers, including applications with LC at critical conditions 133, 134. Nevertheless, SEC has also been applied as ^1^D separation. Examples include SEC × RPLC 135 and SEC × LCCC 135, 136. Readers interested in LC × LC separations of synthetic polymers and oligomers are referred to the review by Uliyanchenko et al. 137.

While Table 3 reflects the strengths and weaknesses of different combinations of retention mechanisms, challenging combinations are not useless. For example, recently a HDC × SEC separation system was developed for the characterization of polymeric nanoparticles 130. The authors combined an aqueous HDC separation of the particles in ^1^D with an organic‐SEC separation in ^2^D, using a mixer and active‐modulation traps to dissolve the particles and to switch from aqueous to organic solvents respectively. The dissolution of the particles by addition of tetrahydrofuran (THF) created good orthogonality (i.e., independence of the two retention axes), because the sample was intrinsically changed. SEC × SEC has been reported as well to study branched polymers 138 and band broadening 139.

#### 3.4.6 Hydrophobic interaction chromatography

HIC finds its sole application in the separation of proteins. HIC is also known as salting‐out chromatography 140. High salt concentrations (e.g., 2 molar ammonium sulfate) are used to promote adsorption of the hydrophobic areas of the protein to the hydrophobic stationary phase 141. Using a gradient toward lower salt concentrations, elution of the proteins is facilitated. The retention increases for buffers with higher molal surface tensions 142. One critical advantage relative to RPLC is that the mobile phase used in HIC typically contains limited amount or no of organic modifiers, conditions that may leave the native structure of the proteins.

Its selectivity is alternative from RPLC 143, 144, which make the technique attractive for 2DLC couplings. However, HIC is not suitable as a ^2^D technique since it requires to slow salt gradients. HIC has only recently come back into fashion and there are few examples of its use in LC × LC. Heinisch et al. used HIC × RPLC–MS for separating antibody–drug conjugates 145, 146.

#### 3.4.7 Chiral chromatography

We refer to chiral chromatography as the collection of LC approaches in which immobilized chiral selectors are used to separate chemical compounds based on one or more centers of chirality in small molecules (<1000 Da). It encompasses a broad spectrum of column chemistries and mobile‐phase combinations. Many bioactive compounds (e.g., pharmaceuticals, agrochemical compounds, amino acids) have one (or more) chiral centers. Other elements of chirality have been described (e.g., helical and topological chirality) and these can be present in larger molecules, but these are not considered here.

A number of chiral selectors can be used to characterize the enantiomeric distribution of small molecules including cyclodextrins, polysaccharides, macrocyclic antibiotics, and Pirkle‐type selectors 147. Polysaccharide derivatives (chiral carbamate/benzoate polymers of cellulose and amylose) are most commonly used, because of their wide applicability range. This class of selectors, which includes several different subtypes, does not cover all the applications. Therefore, chiral molecules are typically screened against different combinations of selectors and mobile phases to find the best candidate for a given molecule. Chiral separations are typically carried out under isocratic conditions, using mobile phases that depend on the type of selector (and, thus, on the chemical groups available for interacting with the analyte) and on the nature of the sample. The most commonly used elution conditions are NPLC and RPLC although IEX and HILIC can be used for certain cases. In recent years chiral supercritical‐fluid chromatography (SFC) has become increasingly popular, due to its high speed, efficiency, and selectivity 148, 149.

Chiral methods have limited resolving power for achiral compounds, are affected by the matrix, and typically require relative long analysis time (>5 min). Therefore, the methods are neither attractive as ^1^D, nor as ^2^D separations in LC × LC. Chiral chromatography has been mainly used as ^2^D separation in heart‐cut approaches 150. However, recently, the introduction of core–shell particles and UHPLC technology and the re‐emergence of SFC have allowed to drastically reduce the time needed to perform this highly‐specific form of chromatography, reducing the analysis time in some cases to less than a second 151, 152. This drastic reduction of the analysis time has made it possible to develop a chiral‐separation method as ^2^D in LC × LC separations. Barhate et al. developed an RPLC × Chiral‐LC and Chiral‐LC × Chiral‐LC methods to study isomers of a synthetic pharmaceutical intermediate 153.

Currently, the lack of a generic chiral‐separation approach (in terms of stationary and mobile phase conditions), capable of distinguishing a broad range of enantiomeric compounds, hinders the development of comprehensive achiral × chiral methods. However, the application of ultrafast chiral separations may lead to interesting developments in the analysis of structurally related compound classes (e.g., chirality analysis of peptides/amino acids 154).

#### 3.4.8 Affinity chromatography

The term affinity chromatography (AfC) has been used in literature to cover different types of studies in which chromatographic selectors have a very specific chemical interaction with sample components with one or a combination of specific molecular features. Typically, proteins are used as immobilized binding agents, because of the specificity of interaction with certain molecules. Examples are antibodies, recognizing specific peptide sequences, but also receptors or other proteins present in biological systems (e.g., HSA), which interact by specific binding sites with small molecules in living organisms, initiating cellular processes or sequestrating these molecules from the matrix environment.

AfC studies may target either only the part of the sample that has interaction with a given target or aim to study the strength of interaction between immobilized binding agent and sample components 155. The first approach typically yields just two (bonded and unbonded) fractions. Examples include protein‐A capturing of antibodies and lecithin capturing of glycoproteins. Titanium‐oxide enrichment of phosphorylated peptides is also sometimes referred to as AfC. These types of separations are more suited to sample‐preparation approaches than to LC × LC. The other alternative, where the separation is driven by different strengths of biomolecular interactions between an immobilized binding agent and sample components, is an interesting and possibly orthogonal separation dimension for LC × LC studies.

Since this type of separation uses mostly immobilized proteins, it is typically performed under conditions that minimize degradation of the ligand and that can be representative of physiological conditions (e.g., isocratic elution using aqueous phosphate buffer at pH 7). However, when MS coupling is needed ammonium‐acetate buffers and low concentrations of organic modifier may be used. To the best of our knowledge, the only publication in which AfC is used in LC × LC is from Hu et al. 156. ^1^D AfC was used to study the interaction between traditional‐Chinese‐medicine components and silica‐bonded HSA. The sample was further separated using an RPLC gradient separation on a C18 silica monolith and coupled with MS. The system presented good orthogonality and allowed the identification of six compounds with different degrees of interaction with HSA.

Although AfC is typically slow and, therefore, has been used as first separation dimension, the progress in speed of analysis brought by monolithic AfC 157 may also allow future application of AfC as ^2^D separation.

#### 3.4.9 Supercritical fluid chromatography

SFC uses mobile phases based on CO_2_, with the addition of organic modifiers, and columns similar to those used in (U)HPLC 158. Strictly speaking, when using modifiers subcritical conditions may prevail. The abbreviation SFC can be thought to imply sub‐ or supercritical‐fluid chromatography. It is potentially faster than LC because of a lower mobile‐phase viscosity and corresponding higher diffusion coefficients of the analytes 159, 160 and it offers good (normal‐phase like) selectivity 158. SFC can be applied to a broad range of low‐molecular‐weight compounds, ranging from nonpolar compounds, such as hydrocarbons and lipids 161, to quite polar molecules, such as pharmaceuticals. One area in which SFC has been quite successful concerns chiral separations 148, 149. So far, SFC has barely been applied to high‐molecular‐weight analytes, probably because of limited solubility in the CO_2_‐based mobile phases.

As seen in Table 3, SFC is potentially a very interesting technique for comprehensive 2D separations. Because SFC allows fast separations, it is potentially most attractive as ^2^D technique. Sarrut et al. 162 described RPLC × SFC for complex mixtures of neutral compounds. SFC is also potentially interesting as ^1^D technique, because the mobile phase is compatible with ^2^D RPLC, as demonstrated by François et al. 163, 164. SFC × SFC using packed (capillary) columns has been demonstrated by Hirata 165, 166. Open‐tubular SFC × SFC 167 is an amazing technological achievement, but not a robust practical approach.

## 4 OPTIMIZATION

### 4.1 Definition

Having established a basic 2D separation through successful development and combination of the individual 1D separations, the analyst faces the decision whether to accept the result or to continue method development through what is typically referred to as optimization. The term optimization is, however, rather vague. It has been widely used to describe different procedures. To judge whether optimization is necessary, a brief discussion regarding the definition and necessity of optimization is useful.

In computer science, optimization often signifies rewriting a program so as to maximize its efficiency and speed. While optimization of LC × LC separations does encompass the pursuit of the best possible system parameters, this does not necessarily connote maximizing performance in terms of quality descriptors, such as peak capacity or orthogonality. Arguably, the motive to develop an LC × LC separation is not the peak capacity itself, but to establish a method to obtain the maximum amount of relevant information on a sample. Enhancing the peak capacity of a method purely for the sake of peak capacity implies maximization rather than optimization. Improving the quality descriptors generally does improve the quality of the separation and, thus, the odds of successfully answering the analytical question, but it is not the most efficient approach for all samples.

The definition of optimization as used in mathematics implies establishing conditions that correspond to the maximum (or minimum) value of a specific, restricted function. In the case of LC × LC separations, such a function should represent the critical information that the method is meant to provide, and is dependent on quality descriptors, such as the peak capacity, which in turn are dependent on the chemical and physical system parameters. Restrictions reflect decisions made at an earlier stage in the method development process (e.g., the use of certain stationary phases) and constraints imposed by the end‐user (e.g., maximum allowable analysis time) and by the system (e.g., pressure‐drop limits).

### 4.2 Decision on the pursuit of optimization

The decision whether to optimize should involve an assessment of the value of the information obtained (i.e., the gain, which depends greatly on the intended purpose of the method) *versus* the additional method‐development time and effort (i.e., the cost, which depends heavily on the optimization strategy applied).

#### 4.2.1 Gain

If the required critical information cannot yet be obtained through the method, optimization is in order. If it can be obtained, then optimization might be unnecessary. This can be illustrated by the separation shown in Fig. 2. The chromatogram shows the separation of several series of industrial surfactants, which all feature a distribution in the length of the propylene oxide (PO) chain. Each observable series represents a difference in (the number of) charged end‐groups. There is a strong correlation between the retention behavior in the ^1^D and ^2^D for each individual series. If the analytical question is satisfactorily answered and the separation is to be applied occasionally to a limited number of samples, it may be decided that further optimization is not necessary. However, if the method is to be applied in a (product or process) control situation, where many samples are to be analyzed and/or the response time is important, then optimization is in order, with the aims of maintaining adequate separation, while minimizing the analysis time, maximizing the sensitivity, and minimizing the solvent consumption.

From the discussion above two types of optimization strategies can be distinguished. Efforts to improve the ability of the method to provide the required information can be denoted as targeted (sample‐dependent) optimization, whereas the application of generic optimization approaches to improve the quality descriptors of the LC × LC method to improve the chances of separating the highest number of sample components can be referred to as general or untargeted (sample‐independent) optimization. Intuitively, the largest gain at the lowest cost makes targeted optimization attractive. Indeed, the specific aim of the optimization here is known. However, untargeted optimization may yield equally good or better results in a shorter time.

#### 4.2.2 The cost

The main cost of LC × LC optimization is an increase in method‐development time. When developing a new LC × LC application, an experienced analyst will rapidly select a reasonable experimental setup. Most of the physical parameters are sample‐independent, they can be optimized following well‐established paths 8, 73. After these hurdles have been taken, LC × LC method development mainly comprises the establishment of the individual separation methods with complementary (“orthogonal”) selectivities and the optimization of the chemical parameters that affect retention and selectivity. If the experience of the analyst is such that the two retention mechanisms are rapidly and efficiently established, optimization remains as the main bottleneck in LC × LC method development. Optimization is never straightforward, if only because of the numerous factors that may be optimized.

**Figure 12:**
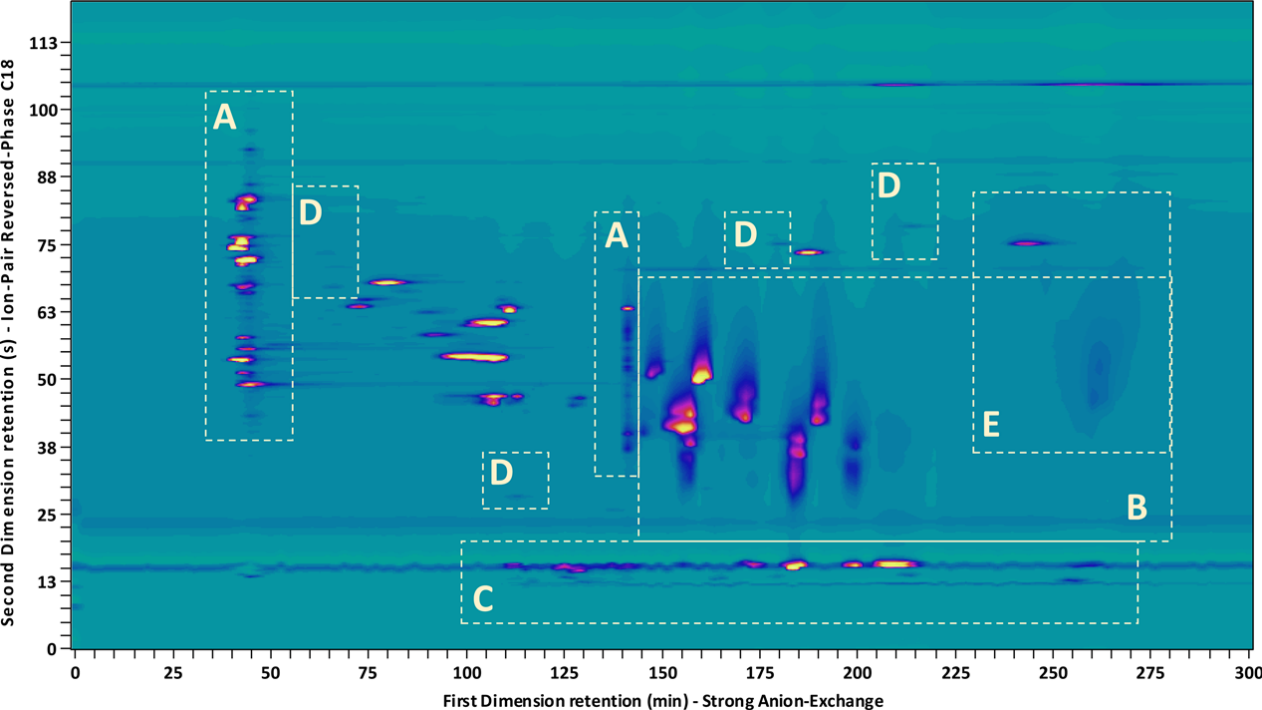
Separation of aged, synthetic dyes by SAX × RP‐DAD. Adapted from 49, with permission

This is illustrated by the separation of a mixture of synthetic dyes shown in Fig. 12 49. Here examples of targeted optimization may encompass (i) improving resolution in desired areas (Fig. 12A), (ii) reducing excessive peak width of charged species to aid quantification (Fig. 12B), (iii) avoiding the occurrence of breakthrough phenomena (Fig. 12C), (iv) improving the sensitivity of the method for analytes present at trace concentrations (Fig. 12D), and/or (v) reducing the analysis time by accelerating late‐eluting compounds (Fig. 12E). In contrast, untargeted optimization of this separation would entail improving the peak capacity and analysis time and enhancing the sensitivity (e.g., through active modulation). Untargeted optimization may alleviate some of the problems encountered in Fig. 12. The gains of untargeted optimization may be equal or higher in the same amount of time as those of targeted optimization, even though specific goals for the latter can be formulated.

### 4.3 Quality descriptors

From the discussion above, it is clear that quality descriptors are needed to describe the quality of the separation. The aim of this review is not to evaluate all approaches for each quality descriptor. Most of the descriptors have been studied and developed extensively. Instead we will limit ourselves to the essential properties and application in optimization.

#### 4.3.1 Orthogonality and surface coverage

A wide range of methods exist to quantify the “orthogonality” (i.e., the degree of dissimilarity between the two separation dimensions), each with their pros and cons. Examples include bin‐counting approaches 44, home‐range theory 168, information theory 169, convex‐hull strategies 170, and the asterisk approach 171. The strengths and weaknesses of most of the different orthogonality metrics was evaluated by Gilar 172 and recently by Schure and Davis 173. Despite the wide selection of metrics, none of them proved to be universally well‐performing. Thus, it was suggested by Schure and Davis that the best approach may comprise the application of a combination of metrics 173.

**Figure 13:**
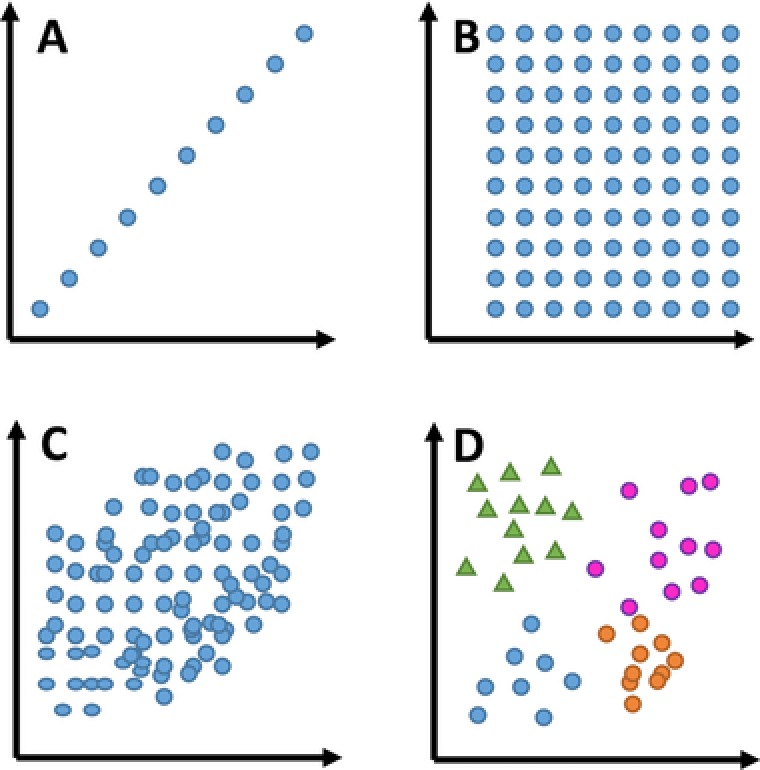
Hypothetical examples of different distributions of peaks across the 2D separation plane in LC × LC separations. In (D), the different colours and shapes represent different classes of compounds separated as in a group‐type separation

From a practical point of view, it is good to realize that the orthogonality of two methods is, in principle, sample dependent. Of course, for any sample, performing two separations with identical selectivities is fruitless (Fig. 13A). ”Full orthogonality” is achieved when the retention mechanisms in the first and ^2^D are statistically unrelated for the sample in question (Fig. 13B). In practice, the two separation dimensions are often found to be slightly correlated (Fig. 13C), but this is not necessarily detrimental for the separation of the analyte mixture. This is illustrated by Fig. 13D, where the hypothetical separation of a mixture containing different classes of compounds is shown. Here, the presence or absence of the triangle‐marked analytes would have a significant impact on the evaluation of the orthogonality of the separation.

With orthogonality as a measure to describe the effectiveness of two retention mechanisms to separate a sample into its constituents and spread the analytes across the separation plane, Rutan et al. argued that a geometric metric (symbolized by *f*_coverage_) is useful to describe the efficient use of the separation space 168. Examples of such metrics include the minimum‐convex‐hull method 168 and the modified 16 or unmodified 17 ecological home‐range theory.

#### 4.3.2 Effective peak capacity

The peak capacity is arguably one of the most important quality descriptors in developing separation methods for complex samples. The peak capacity represents the maximum number of separated singlet peaks that fit within the separation space, the peak capacity gives a good indication on the potential separation power of the method. Accurate prediction of the peak capacity depends on factors such as the retention mechanism and the used elution mode 174, 175. For gradient elution, calculation of the peak capacity is particularly simple because the peaks generally have roughly equal widths, resulting in Eq (1) with

(1) nc=1+tR, last −tR, first W,

here, *W* represents the average width of the peak at the baseline (equal to four times the SD; W= 4*σ*). The fundamental advantage of comprehensive 2D chromatography is that peak capacities of the ^1^D and ^2^D may be multiplied

(2) nc,2D∗=1nc2ncf coverage β.

To correct n_c,2D_ for undersampling (see Section 2.1) due to the modulation process, Davis et al. 176 developed an average peak‐broadening factor (*β*)

(3) $$\left\langle \beta \right\rangle =\sqrt{1+3.35{\left(\frac{t_{s}}{{}^{1}W}\right)}^{2}},$$

where $t_{s}$ is the modulation time and ^1^ *W* represents the (average) ^1^D peak width.

Vivó‐Truyols et al. 31 proposed a rigorous equation for the peak capacity for LC × LC separations using gradients in both dimensions.

(4) nc,2D=1tG4Rs1σ peak 2+2t mod 2δ det 22tG4Rs2σ peak 2+1F2F22t mod 2δ inj 2,

Here, σ_peak_ represents the average SD, *F* the flow rate, *t*_G_ the length of the gradient, *t*_mod_ the modulation time, and *R*_s_ the desired resolution between two peaks (typically a value of 1 is used). δ inj 2 is a parameter related to the injection system. It ranges between 12 and 4, although typically a value of 4 is used 36. δ det 2 is a detection factor (also between 12 and 4). Using the statistical overlap theory, Davis et al. found an experimental value for δ det 2 of 4.76 176. Equation 4 accounts for both the effect of undersampling and that of injection band broadening in the ^2^D.

Some authors opt to correct the peak capacity for the incomplete coverage of the separation space, using the fractional coverage *f*_coverage_. While correcting for undersampling, this yields 16

(5) nc,2D∗=1nc·2nc·f coverage β.

This “effective peak capacity” is no longer a characteristic of the separation system, because the value depends greatly on the sample.

#### 4.3.3 Dilution factors

From the perspective of analyte detectability, it is important to consider dilution factors to assess potential detection problems. The dilution factor (DF) in the ^1^D can be calculated from

1DF=2π1σ peak 1F1V inj ,

with ^1^σ_peak_ depicting the average SD of the peak in the ^1^D, ^1^ *F* the ^1^D flow rate, and ^1^ *V*_inj_ the injection volume. For calculation of the ^2^D dilution factor, the total band broadening in the ^2^D needs to be taken into account given by 31

(6) 2σ total =2σ peak 2+1F2F21FF22tA2δ inj 2,

here, ^2^ *t*_A_ denotes the analysis time in the ^2^D and *FF* represents the focusing factor which accounts for contraction of the transferred volume (^2^D injection volume) due to focusing at the inlet of the ^2^D column or on a trap cartridge in the loop. The focusing factor is given by 43

(7) $$FF=\frac{{}^{2}k_{1e}+1}{{}^{2}k_{2e}+1},$$

where ^2^ *k*_1e_ represents the retention factor in the ^2^D in the presence of the ^1^D mobile phase (or, in case the composition is altered during modulation, the ^2^D “loading solvent”) and ^2^ *k*_2e_ depicts the ^2^D retention factor in the presence of the ^2^D mobile phase. Using these relations, the dilution factor in the ^2^D can be calculated asThe total dilution in the LC × LC system is the product of the ^1^D and ^2^D dilution factors (^2D^ *DF* = ^1^ *DF* × ^2^ *DF*).

#### 4.3.4 Other quality descriptors

Another useful descriptor used to assess the separation is the resolution between the peaks. Several strategies exist to calculate the 2D resolution, but most of these require a full description of each (partially overlapped) peak, which is not typically available. Approaches involving valley‐to‐peak ratios circumvent this problem and a metric was proposed by Schure for Gaussian‐shaped peaks 177. Peters et al. used the saddle‐point concept to calculate the valley‐to‐peak ratios for experimental peaks which are not of Gaussian shape 178 based on their peak detection algorithm 179.

Finally, the analysis time of the method is an obvious quality descriptor to take into account. While the total analysis time is equal to the ^1^D analysis time, ^1^ *t*_A_, it is good to realize that ^1^ *t*_A_ is strongly dependent on the analysis time of the ^2^D^2^ *t*_A_, which determines the modulation time *t*_mod_. Since the majority of LC × LC methods utilize gradient elution in the ^2^D, it is not surprising that the main factor governing ^2^ *t*_A_ (and indirectly, ^1^ *t*_A_) is the ^2^D gradient. Sarrut and co‐workers pointed out that the ^2^D column equilibration and dwell volume play a significant role in the optimization of the total analysis time 35.

### 4.4 Optimization Approaches

As previously discussed, optimization implies seeking a balance between (i) achieving sufficient separation (ii) at the lowest possible expense. What is seen as “sufficient separation” is determined by the requirements that follow from the analytical question. If only a few peaks are of interest (e.g., targeted analysis of specific compounds) complete resolution of these peaks may be required. For a very complex sample, containing an endless number of relevant components, full orthogonality, and a high peak capacity may be strived for. In between these two extremes lie other practical examples, such as the group‐type separation of a number of classes (e.g., lipids, detergents). The lowest possible expense refers to minimizing the total analysis time, dilution factors, and/or eluent volumes, but also maximizing the efficiency through optimal use of parameters including the operating pressure, column lengths, and particle diameters.

The conventional way to seek a good compromise is experimental (“trial‐and‐error”) optimization where the analyst manually varies method parameters until the resulting separation delivered by the method is found acceptable. While potentially applicable for targeted optimization 91, such iterative modifications are rather cumbersome and extremely time consuming. Also, the true (“global”) optimum is unlikely to be found and sub‐optimal conditions are likely be established, resulting in a waste of resources (time, solvents, columns, instrumentation), especially if the LC × LC method is to be applied numerous times. In most cases, “trial‐and‐error” optimization is conducted using the univariate (“one‐factor‐at‐a‐time”) approach, establishing the optimum value for one parameter, while keeping all other conditions constant. While this approach seems practical, it is an ill‐advised strategy if the quality of the separation is affected by multiple interdependent parameters.

**Figure 14:**
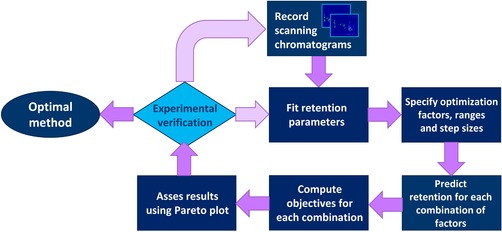
Schematic representation of an algorithmic optimization procedure. Reproduced from 187 with permission

To overcome the daunting problem of method development in LC and, especially, LC × LC chromatographers have attempted to implement predictive tools and optimization algorithms to establish optimal conditions in a much more efficient manner. Generally, these algorithms use retention modeling for each compound to predict the chromatographic separation of the analyte mixture under various conditions, after which quality descriptors are used to select the most favorable option of the predicted separations. In principle, the algorithmic procedure follows a protocol that can be divided in a number of steps (Fig. 14).

**Figure 15:**
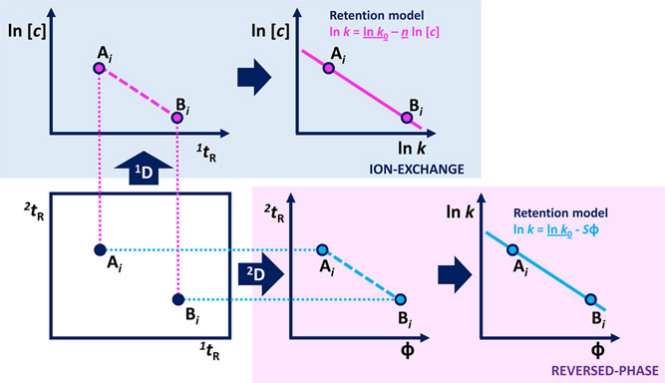
Schematic illustrations of using two scanning LC × LC experiments with (isocratic) conditions A and B. Two sets of retention times of compound *i* are obtained. The retention times can then be expressed as retention factors, allowing the retention parameters (ln*k*_0_ and *n* for IEX and ln*k*_0_ and *S* for RP) to be determined

First, initial chromatographic data on the analyte mixture is required to allow retention models to be established (Fig. 14). Several so‐called scouting experiments are carried out utilizing isocratic or gradient conditions. The number of scanning experiments required depends on the retention model used. For two‐parameter models, two scanning runs may suffice (e.g., linear‐solvent strength 180 and adsorption model 181), whereas three‐parameter models (e.g., quadratic 182, mixed mode 183 and Neue–Kuss 184) require more experiments. By performing several experiments at sufficiently different conditions, the obtained retention times can be used to fit the retention parameters for each compound. In the case of two scanning runs, this comes down to solving two equations for two unknowns. The schematic in Fig. 15 illustrates the procedure for isocratic elution, but gradients are often more appropriate and the same strategy can be applied with mathematical conversion. For some models, the retention model results in a gradient equation that is difficult to integrate, so that approximations are necessary. Linear models and the empirical model developed by Neue and Kuss 184 can be integrated easily. For scanning gradients to be utilized effectively for retention‐parameter determination, it is important that the slopes of the gradient of the two scouting experiments differ by a factor of three or more 185, 186.

Having determined the retention parameters for both dimensions for all analytes of interest, the algorithm can predict the retention time for a theoretically endless number of chromatographic conditions. To limit the number of methods to be evaluated, software packages can allow the analyst to specify optimization domains, based on which the algorithm can generate methods to be evaluated. For example, the user could decide to investigate the effect of the initial and final modifier mobile phase fraction (*φ*_init_ and *φ*_final_, respectively) of the gradient in the ^2^D. Setting the investigated domains for *φ*_init_ to a range from 0.05 to 0.3 and for *φ*_final_ from 0.7 to 1.0, each with steps of 0.05, the algorithm can try all possible linear combinations and investigate a total of 42 LC × LC methods. Considering other selectivity‐related chemical parameters, such as the time before the gradient is programmed to start, the length of the gradient, but also more complex gradient assemblies, such as segmented and shifting gradients, the number of chromatographic conditions to test can rise dramatically.

**Figure 16:**
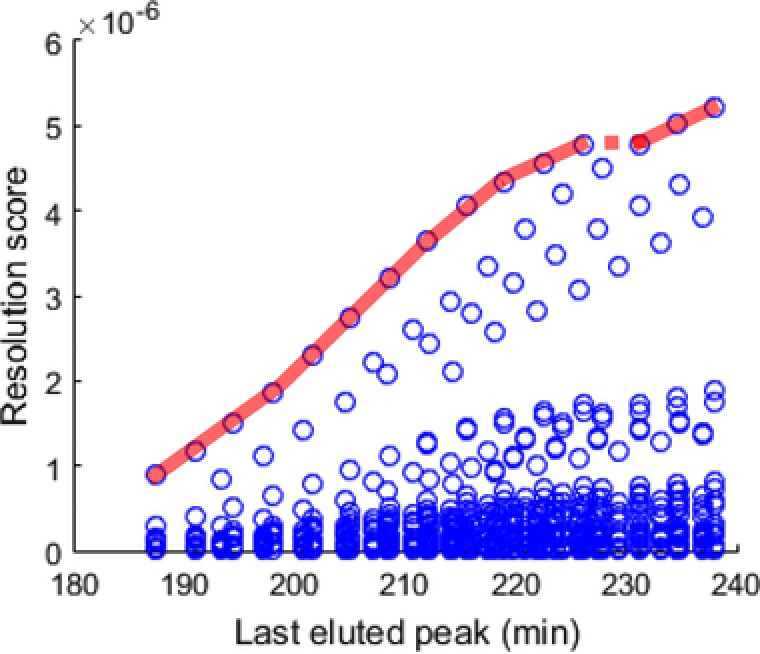
Example of a Pareto‐optimality (PO) plot with each point representing one of the 10 368 evaluated simulated LC × LC separations of a mixture of aged, synthetic dyes. The PO front (in red) reflects all PO points in the plot. For these points the two plotted criteria may not both be improved simultaneously. See Supporting Information S3 for a more detailed clarification on the chromatographic conditions, analyte mixture, and the optimization parameters used to arrive at this PO‐plot. Plot created using the Program for Interpretive Optimization of 2D Resolution (PIOTR) 187

With the retention times predicted for all LC × LC methods evaluated by the algorithm, the optimal method(s) must be found. To accomplish this, the algorithm can be instructed to calculate the quality descriptors (see section 4.2) for each predicted separation and present the results in a Pareto‐optimality (PO) plot. An example of a PO‐plot is provided in Fig. 16 in which the quality descriptors 2D resolution 177 (see Supporting Information Section S5 for more information) and analysis time were plotted against each other for 10 368 predicted LC × LC methods for the separation of aged, synthetic dyes 187 (see Supporting Information S5 for a full description of how the PO‐plot was established). It can be seen that the majority of the simulated methods yield sub‐optimal resolution and the PO front (indicated in red) allows efficient selection of the methods that provide the highest resolution within the specified optimization ranges. Many different quality descriptors can be plotted in such PO plots.

A crucial aspect of using predictive tools is experimental verification to validate the established retention parameters. For example, for HILIC it is known that the stationary and mobile phase, significantly influence the type of interactions between analyte and stationary‐phase packing 105, 188. As a result, the selected retention model may not adequately describe the retention behavior, resulting in incorrect predictions. It is thus prudent to, either during the early or final stages of the algorithmic optimization process, verify the prediction of one or more LC × LC separations that were not used by the algorithm to determine the retention parameters.

In the event that the experimental verification proves unsatisfactory, a different retention model may be used to restart the algorithmic procedure, as is reflected in the flow chart shown in Fig. 14. Conversely, if a satisfactory‐optimal method is found, the analyst may opt to end the optimization process. However, in some cases the optimal method may allow the separation and detection of additional, previously co‐eluting, species so that the analyst can reinitiate the (evaluation of the) scanning experiments using the optimal method as starting point. Peak‐detection algorithms may allow this process to iterate automatically until a satisfactory optimum is found.

## 5 CONCLUDING REMARKS

**Figure 17:**
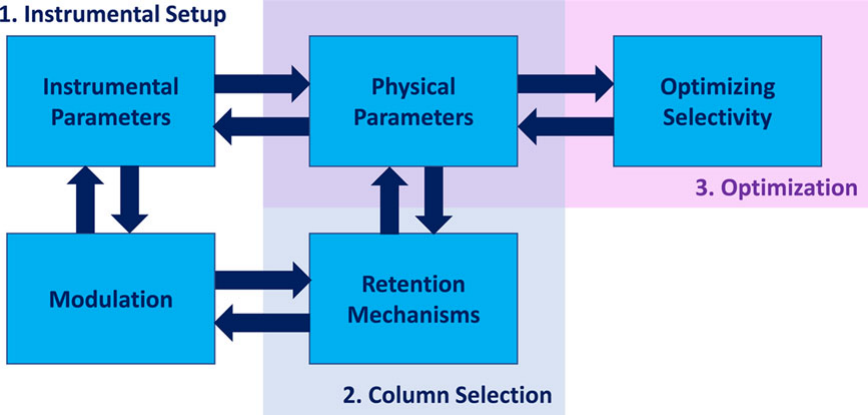
Schematic representation of the main processes involved in developing and optimizing LC × LC separations

Instrumentation for LC × LC has advanced greatly in recent years, robust and reliable instrumentation is available and loop‐type modulators are well established. This allows LC × LC methods to be developed by a sensible selection of two highly different 1D retention mechanisms and accompanying columns. Physical parameters (column diameters, flow rates, and particle sizes) can be optimized so as to achieve the highest possible performance (peak capacity) in the shortest possible time. This often results in impressive 2D separations. However, such separations can usually be improved further by adapting some of the many instrumental parameters available, including different ^2^D gradients for every collected fraction. The number of parameters is such that finding the overall (global) optimum has become a daunting task. To realize LC × LC separations that are effective in terms of time, sensitivity, and solvent consumption, the use of systematic optimization procedures is indispensable. The main processes involved in developing and optimizing LC × LC separations are summarized in Fig. 17. Method development and optimization efforts in LC × LC may be greatly reduced by a systematic approach and user‐friendly software. This will gradually remove one of the major obstacles to the proliferation of LC × LC.

## Supporting information

Supporting information
