# Supplementary material for: Optimizing separations in online comprehensive two‐dimensional liquid chromatography
Source: J Sep Sci. 2017 Nov 23;41(1):68–98. doi: 10.1002/jssc.201700863 (PMC5814945; doi:10.1002/jssc.201700863)
Supplement: Supplementary file 1 — Supporting information [file JSSC-41-68-s001.pdf]

SUPPORTING INFORMATION

Optimizing Separations in On-line Comprehensive Two-dimensional Liquid Chromatography

Review

Bob W.J. Pirok[1,2,*], Andrea F.G. Gargano[1,3], Peter J. Schoenmakers[1]

[1] University of Amsterdam, van 't Hoff Institute for Molecular Sciences, Analytical-Chemistry Group, Science Park 904, 1098 XH Amsterdam, The Netherlands.
[2] TI-COAST, Science Park 904, 1098 XH Amsterdam, The Netherlands
[3] Vrije Universiteit Amsterdam, Department of Bioanalytical Chemistry, Amsterdam Institute for Molecules, Medicines and Systems, de Boelelaan 1085, 1081HV Amsterdam, The Netherlands

(*) Corresponding author
E-mail: B.W.J.Pirok@uva.nl

Table of Contents

S-1 Separation of industrial surfactant mixture 3
S-2 Tailored Shifting Gradient 4
S-3 Separation of mixture of sulfonated ethoxylates 4
S-4 Overview of references and applications for different combinations of retention mechanisms 5
S-5 Pareto-optimality plot 6
References 9

S-1 Separation of industrial surfactant mixture

For the separation of industrial surfactants as shown in Figure 2 of the review. A Shimadzu LC20 setup was used. The system consisted of a CBM-20A communications bus module which controlled the system, four LC20AD pumps, a SIL-20AC autosampler, a DGU-20A degasser and a CTO-20A column oven. Inside the column oven a two-position Cheminert 10-port valve by Valco Instruments Co. Inc. and was located and configured for comprehensive operation.

Dimension 1
Column: Acclaim™ Surfactant Plus (250 mm × 2.1 mm, particle size: 3 µm). Gradient analysis. Oven temperature: 40°C. Flowrate: 10 µL min^{-1} at 0.0 min, 80 µL min^{-1} at 0.1 min, 80 µL min^{-1} at 6.2 min, 10 µL min^{-1} at 6.3 min. Analysis time: 201 minutes. Mobile phase: acetonitrile (A) and 0.1 M ammonium acetate in water with pH 5.4 through addition of glacial acetic acid (B). Gradient: 60% B at 0.0 min, 20% B at 130 min, 20% B at 140.0 min, 60% B at 150.0 min, 60% B at 201 min. Injection volume: 20 µL.

Dimension 2
Column: Phenomenex Kinetex C18 (50 mm × 4.6 mm, particle size: 5 µm). Gradient analysis. Oven temperature: 40 °C, flowrate: 3.5 mL min^{-1}, analysis time: 3 minutes. Mobile phase: water/acetonitrile [95:5] (A) and acetonitrile/water [95:5] (B). Gradient: 0% B at 0 sec, 100% B at 135 sec, 0% B at 165 sec, 0% at 180 sec.

S-2 Tailored Shifting Gradient

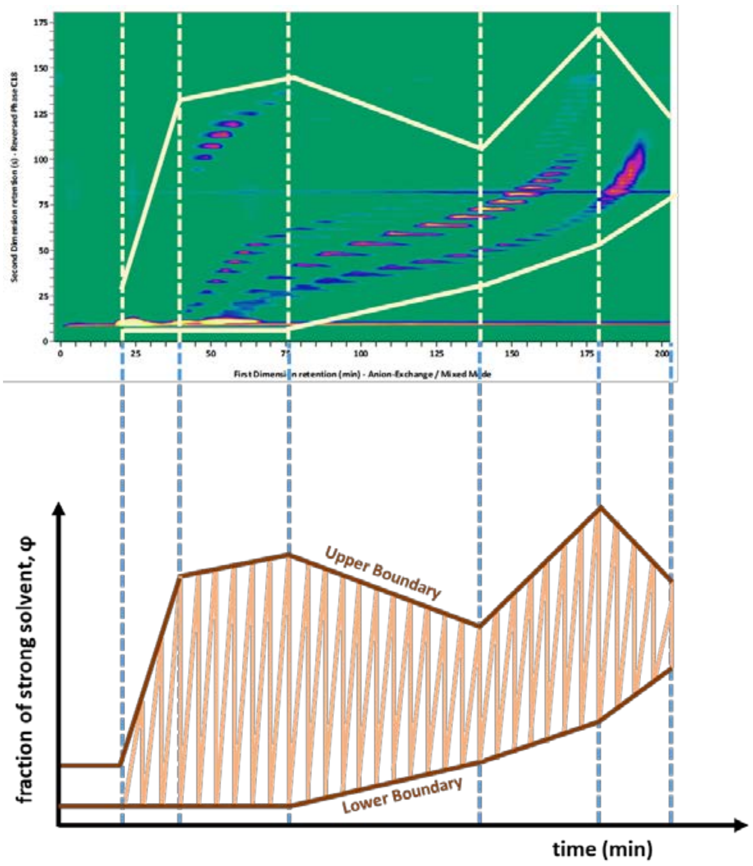

Figure S-1 – Example of a tailored shifting gradient program proposed to optimize the use of the separation space. Note that all the boundary lines connect to avoid peaks being split between dimensions.

S-3 Separation of mixture of sulfonated ethoxylates

Agilent PL-SAX (150 mm × 2.1 mm, particle size: 8 µm). Isocratic analysis. Oven temperature: 40 ^{o}C, Flowrate: 1.5 mL min^{-1}, Analysis time: 60 minutes. Mobile phase: 10 mM ammonium acetate in water (A), 10 mM ammonium acetate in acetonitrile/water [1:1] (B). Injection volume: 10 µL.

S-4 Overview of references and applications for different combinations of retention mechanisms

Table S-1 - Overview of references and applications for different combinations of retention mechanisms.

	^{2}RP	^{2}NP	^{2}HILIC	^{2}HIC	^{2}IEC	^{2}SEC-Aq	^{2}SEC-Or	^{2}Ag	^{2}Chiral	^{2}Affinity	^{2}SFC
^{1}RP	[1–3]	[4]	[5]				[6,7]	[8]	[9]		[2]
^{1}NP	[4,10–13]						[14,15]				
^{1}HILIC	[16–18]		[19,20]		[21]						
^{1}HIC	[22–24]										
^{1}IEC	[25,26]				[27]	[28]					
^{1}SEC-A	[29]						[30]				
^{1}SEC-O	[31]						[32,33]				
^{1}Ag	[34–37]										
^{1}Chiral									[9]		
^{1}Affinity	[38]										
^{1}SFC	[39]										[40]

S-5 Pareto-optimality plot

The scanning experiments comprised the separation of a mixture of aged, synthetic dyes by strong anion-exchange and ion-pair reversed-phase chromatography [41] using scouting conditions as reported by Pirok and co-workers [42].

To establish the retention parameters of all selected analytes of interest, the adsorption model [43] was used to fit the ion-exchange data and the linear solvent-strength model [44] was used to fit the reversed-phase data. The MATLAB-based PIOTR program (Program for Interpretive Optimization of Two-dimensional Resolution) used the FMINSEARCH function of MATLAB to solve the two integrated gradient equations for two unknown parameters in each dimension to determine the retention parameters for each compound. The resulting retention parameters are shown in Table S-2. For the ion-exchange separation, it can be seen that unrealistic retention parameters were found for analytes 1 to 14. This can be explained from the absence of retention for these analytes under ion-exchange conditions. The model failed at describing the non-existent retention behaviour. Moreover, for the retained analytes 36 and 28, unrealistic parameters were found in ion-exchange and reversed-phase LC, respectively. This shows that it is prudent to inspect the obtained retention parameters and improve or discard them if necessary.

Table 1 also lists the Akaike Information Criterion (AIC) [45] values for each compound. This goodness-of-fit value provides an indication on how well the used retention model is able to describe the retention behaviour. These goodness-of-fit values were earlier obtained by using five experimental datapoints per compound instead of two. Generally, lower values reflect a better ability of the retention model to describe the retention. These, and other statistical indications may help to assess the performance of the used retention model, as was also shown for the assessment of retention models to describe HILIC retention by Cesla and co-workers [46].

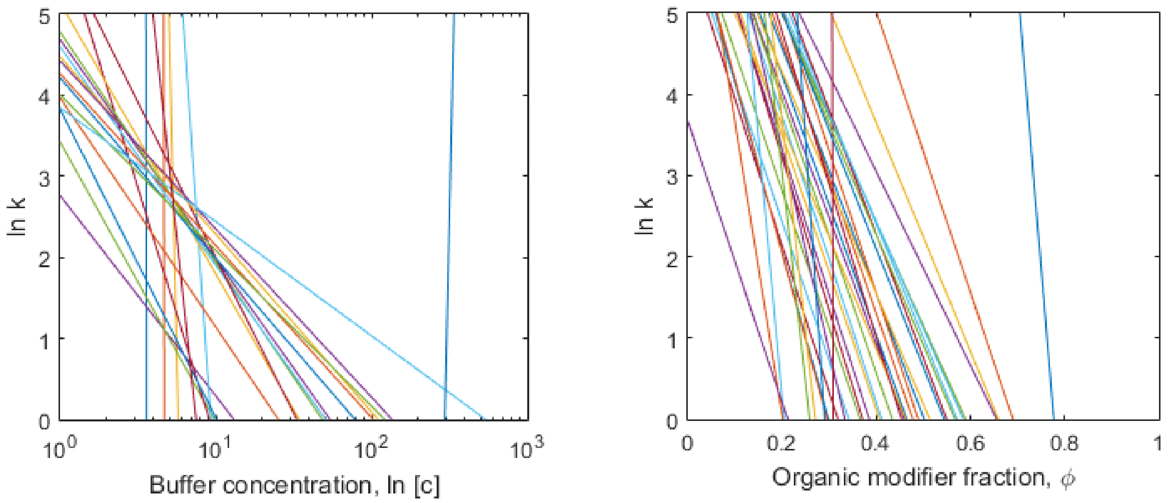

Figure S-2 – Established retention plots of 34 analytes for ion-exchange (left) using the adsorption model and ion-pair reversed-phase (right) using the linear solvent-strength model.

Table S-2 – Overview of fitted retention parameters all analytes of interest in both dimensions.

	ION-EXCHANGE			***REVERSED-PHASE***		
**Analyte**	$\ln k_0$	n	**AIC**	$\ln k_0$	S	**AIC**
1	-207.25	-36.44	17.44	53.88	69.39	-61.99
2	-207.25	-36.44	17.62	11.94	17.31	-52.45
3	-207.25	-36.44	16.79	9.28	14.08	-51.21
4	-207.25	-36.44	13.83	7.81	11.93	-48.64
5	-207.25	-36.44	13.37	7.56	12.83	-50.66
6	-207.25	-36.44	13.23	8.00	14.01	-52.00
7	-207.25	-36.44	16.32	8.37	15.24	-49.16
8	-207.25	-36.44	15.25	7.60	15.16	-53.49
9	-207.25	-36.44	17.35	8.53	17.46	-52.51
10	-207.25	-36.44	14.58	6.88	16.91	-50.64
11	-207.25	-36.44	16.78	6.91	17.95	-53.62
12	-207.25	-36.44	13.30	6.21	17.01	-54.76
13	-207.25	-36.44	13.71	5.87	17.18	-56.34
14	-207.25	-36.44	18.31	5.74	18.05	-54.03
17	63.34	36.30	-53.40	7.00	13.63	-50.44
18	2.76	1.07	-28.14	7.14	15.57	-51.19
19	3.42	1.50	-30.63	7.29	16.82	-53.03
20	26.94	12.10	-42.41	7.25	17.63	-53.21
21	5.97	2.71	-33.82	6.99	18.85	-51.20
22	3.82	1.65	-31.10	6.23	20.90	-53.45
23	3.96	1.23	-25.57	6.32	21.39	-54.18
24	5.14	1.46	-25.73	6.36	13.75	-49.90
25	4.68	1.17	-22.72	8.31	24.93	-55.09
26	4.78	1.23	-27.62	13.43	52.02	-60.94
27	4.59	1.17	-26.67	12.95	63.45	-62.43
28	5.83	1.67	-25.61	838.51	2722.60	-1.84
29	4.21	0.97	-25.03	24.73	85.32	-64.01
30	4.26	0.92	-23.62	7.25	36.02	-56.10
31	4.47	0.95	-25.15	14.16	52.41	-60.45
32	4.42	0.90	-22.61	3.66	17.30	-52.14
33	4.00	0.83	-23.53	7.71	13.60	-50.70
34	3.85	0.61	-16.02	7.70	13.18	-53.50
35	15.77	7.84	-42.20	8.53	18.81	-50.67
36	-207.25	-36.44	14.14	7.92	14.56	-51.02

Using the obtained retention parameters, potential LC×LC methods were generated by varying several method parameters as is reflected in Table S-3. The total number of simulated LC×LC methods was thus 10,368 (product of all options).

Table S-3 – Optimization factors, ranges and values used for optimization.

Method Parameter	*Min. Value*	*Max. Value*	*Increments*	*# Options*
^{1}D initial buffer conc. $[c]_{init}$ (mM)	0	10	5	3
^{1}D final buffer conc. $[c]_{final}$ (mM)	25	100	25	4
^{1}D gradient time (min)	40	150	10	12
^{2}D initial modifier fraction φ_{init}	0.05	0.3	0.05	6
^{2}D initial modifier fraction φ_{final}	0.7	0.95	0.05	6
^{2}D gradient time (min)	1.3	1.5	0.2	2

The retention times were predicted for each of the generated methods and the quality descriptor resolution was calculated according to Schure's metric for resolution in two-dimensional chromatography [47]. The resolutions between all the peaks were summarized into a Deringer-based score [42]. The resulting resolution values were then plotted against the analysis time (time for the last analyte to elute) in the Pareto-optimality plot as shown below.

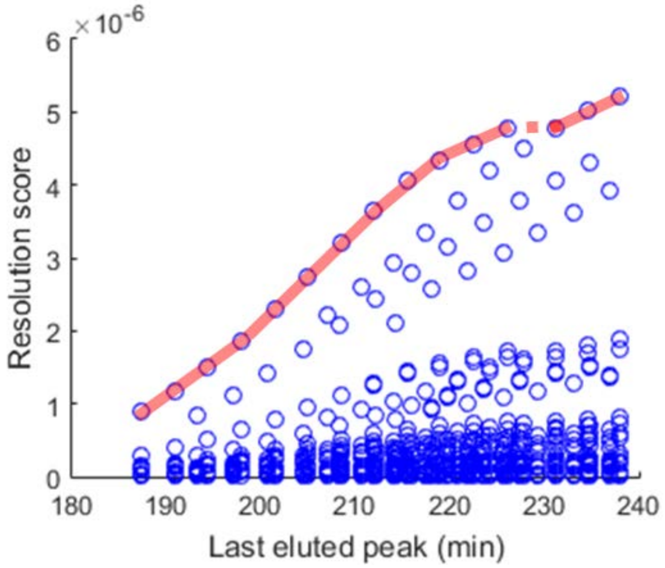

Figure S-3 – Pareto-optimality plot comprising the resolution plotted against the analysis time for 10368 simulated LC×LC methods.

References

[1] Donato, P., Cacciola, F., Sommella, E., Fanali, C., Dugo, L., Dachà, M., Campiglia, P., Novellino, E., Dugo, P., Mondello, L., Online comprehensive RPLC × RPLC with mass spectrometry detection for the analysis of proteome samples. *Anal. Chem.* 2011, 83, 2485–2491.

[2] Sarrut, M., Corgier, A., Crétier, G., Le Masle, A., Dubant, S., Heinisch, S., Potential and limitations of on-line comprehensive reversed phase liquid chromatography×supercritical fluid chromatography for the separation of neutral compounds: An approach to separate an aqueous extract of bio-oil. *J. Chromatogr. A* 2015, 1402, 124–133.

[3] Sandra, K., Vanhoenacker, G., Vandenheede, I., Steenbeke, M., Joseph, M., Sandra, P., Multiple heart-cutting and comprehensive two-dimensional liquid chromatography hyphenated to mass spectrometry for the characterization of the antibody-drug conjugate ado-trastuzumab emtansine. *J. Chromatogr. B* 2016, 1032, 119–130.

[4] Jandera, P., Fischer, J., Lahovská, H., Novotná, K., Česla, P., Kolářová, L., Two-dimensional liquid chromatography normal-phase and reversed-phase separation of (co)oligomers. *J. Chromatogr. A* 2006, 1119, 3–10.

[5] Holčapek, M., Ovčačíková, M., Lísa, M., Cífková, E., Hájek, T., Continuous comprehensive two-dimensional liquid chromatography-electrospray ionization mass spectrometry of complex lipidomic samples. *Anal. Bioanal. Chem.* 2015, 407, 5033–5043.

[6] Uliyanchenko, E., Cools, P. J. C. H., Van Der Wal, S., Schoenmakers, P. J., Comprehensive two-dimensional ultrahigh-pressure liquid chromatography for separations of polymers. *Anal. Chem.* 2012, 84, 7802–7809.

[7] Greiderer, A., Steeneken, L., Aalbers, T., Vivó-Truyols, G., Schoenmakers, P., Characterization of hydroxypropylmethylcellulose (HPMC) using comprehensive two-dimensional liquid chromatography. *J. Chromatogr. A* 2011, 1218, 5787–5793.

[8] Dugo, P., Favoino, O., Tranchida, P. Q., Dugo, G., Mondello, L., Off-line coupling of non-aqueous reversed-phase and silver ion high-performance liquid chromatography-mass spectrometry for the characterization of rice oil triacylglycerol positional isomers. *J. Chromatogr. A* 2004, 1041, 135–142.

[9] Barhate, C. L., Regalado, E. L., Contrella, N. D., Lee, J., Jo, J., Makarov, A. A., Armstrong, D. W., Welch, C. J., Ultrafast Chiral Chromatography as the Second Dimension in Two-Dimensional Liquid Chromatography Experiments. *Anal. Chem.* 2017, 89, 3545–3553.

[10] Tian, H., Xu, J., Guan, Y., Comprehensive two-dimensional liquid chromatography (NPLC×RPLC) with vacuum-evaporation interface. *J. Sep. Sci.* 2008, 31, 1677–1685.

[11] Divito, E. B., Kroniser, K. M., Cascio, M., Multidimensional Liquid Chromatography Coupled with Tandem Mass Spectrometry for Identification of Bioactive Fatty Acyl Derivatives. *Front. Physiol.* 2016, 7, 608.

[12] Cacciola, F., Farnetti, S., Dugo, P., Marriott, P. J., Mondello, L.,

Comprehensive two-dimensional liquid chromatography for polyphenol analysis in foodstuffs. *J. Sep. Sci.* 2017, 40, 7–24.

[13] Dugo, P., Favoino, O., Luppino, R., Dugo, G., Mondello, L., Comprehensive Two-Dimensional Normal-Phase (Adsorption)−Reversed-Phase Liquid Chromatography. *Anal. Chem.* 2004, 76, 2525–2530.

[14] Kok, S. J., Hankemeier, T., Schoenmakers, P. J., Comprehensive two-dimensional liquid chromatography with on-line Fourier-transform-infrared-spectroscopy detection for the characterization of copolymers. *J. Chromatogr. A* 2005, 1098, 104–110.

[15] Graef, S. M., Van Zyl, A. J. P., Sanderson, R. D., Klumperman, B., Pasch, H., Use of gradient, critical, and two-dimensional chromatography in the analysis of styrene- and methyl methacrylate-grafted epoxidized natural rubber. *J. Appl. Polym. Sci.* 2003, 88, 2530–2538.

[16] Willemse, C. M., Stander, M. A., Vestner, J., Tredoux, A. G. J., de Villiers, A., Comprehensive Two-Dimensional Hydrophilic Interaction Chromatography (HILIC) × Reversed-Phase Liquid Chromatography Coupled to High-Resolution Mass Spectrometry (RP-LC-UV-MS) Analysis of Anthocyanins and Derived Pigments in Red Wine. *Anal. Chem.* 2015, 87, 12006–15.

[17] Kalili, K. M., De Smet, S., Van Hoeylandt, T., Lynen, F., De Villiers, A., Comprehensive two-dimensional liquid chromatography coupled to the ABTS radical scavenging assay: A powerful method for the analysis of phenolic antioxidants. *Anal. Bioanal. Chem.* 2014, 406, 4233–4242.

[18] Gargano, A. F. G., Duffin, M., Navarro, P., Schoenmakers, P. J., Reducing Dilution and Analysis Time in Online Comprehensive Two-Dimensional Liquid Chromatography by Active Modulation. *Anal. Chem.* 2016, 1, 1–16.

[19] Wang, Y., Lu, X., Xu, G., Development of a comprehensive two-dimensional hydrophilic interaction chromatography/quadrupole time-of-flight mass spectrometry system and its application in separation and identification of saponins from Quillaja saponaria. *J. Chromatogr. A* 2008, 1181, 51–59.

[20] Deguchi, K., Keira, T., Yamada, K., Ito, H., Takegawa, Y., Nakagawa, H., Nishimura, S. I., Two-dimensional hydrophilic interaction chromatography coupling anion-exchange and hydrophilic interaction columns for separation of 2-pyridylamino derivatives of neutral and sialylated N-glycans. *J. Chromatogr. A* 2008, 1189, 169–174.

[21] Schneider, S., Naegele, E., Krieger, S., Online 2D-LC Analysis of Complex N-Glycans in Biopharmaceuticals Using the Agilent 1290 Infi nity 2D-LC Solution. *Appl. Note Agil. 5991-5349EN.pdf* n.d.

[22] Sarrut, M., Corgier, A., Fekete, S., Guillarme, D., Lascoux, D., Janin-Bussat, M.-C., Beck, A., Heinisch, S., Analysis of antibody-drug conjugates by comprehensive on-line two-dimensional hydrophobic interaction chromatography x reversed phase liquid chromatography hyphenated to high resolution mass spectrometry. I − Optimization of separation conditions. *J. Chromatogr. B* 2016, 1032, 103–111.

[23] Sarrut, M., Fekete, S., Janin-Bussat, M. C., Colas, O., Guillarme, D., Beck, A.,

Heinisch, S., Analysis of antibody-drug conjugates by comprehensive on-line two-dimensional hydrophobic interaction chromatography x reversed phase liquid chromatography hyphenated to high resolution mass spectrometry. II-Identification of sub-units for the characteri. *J. Chromatogr. B Anal. Technol. Biomed. Life Sci.* 2016, 1032, 91–102.

[24] Birdsall, R. E., Shion, H., Kotch, F. W., Xu, A., Porter, T. J., Chen, W., A rapid on-line method for mass spectrometric confirmation of a cysteine-conjugated antibody-drug-conjugate structure using multidimensional chromatography. *MAbs* 2015, 7, 1036–1044.

[25] Vonk, R. J., Gargano, A. F. G., Davydova, E., Dekker, H. L., Eeltink, S., de Koning, L. J., Schoenmakers, P. J., Comprehensive Two-Dimensional Liquid Chromatography with Stationary-Phase-Assisted Modulation Coupled to High-Resolution Mass Spectrometry Applied to Proteome Analysis of Saccharomyces cerevisiae. *Anal. Chem.* 2015, 87, 5387–5394.

[26] Pirok, B. W. J., Knip, J., van Bommel, M. R., Schoenmakers, P. J., Characterization of synthetic dyes by comprehensive two-dimensional liquid chromatography combining ion-exchange chromatography and fast ion-pair reversed-phase chromatography. *J. Chromatogr. A* 2016, 1436, 141–146.

[27] Shellie, R. A., Tyrrell, E., Pohl, C. A., Haddad, P. R., Column selection for comprehensive multidimensional ion chromatography. *J. Sep. Sci.* 2008, 31, 3287–3296.

[28] Bushey, M. M., Jorgenson, J. W., Automated instrumentation for comprehensive two-dimensional high-performance liquid chromatography of proteins. *Anal. Chem.* 1990, 62, 161–167.

[29] Opiteck, G. J., Jorgenson, J. W., Anderegg, R. J., Two-Dimensional SEC/RPLC Coupled to Mass Spectrometry for the Analysis of Peptides. *Anal. Chem.* 1997, 69, 2283–2291.

[30] Pirok, B. W. J., Abdulhussain, N., Aalbers, T., Wouters, B., Peters, R. A. H., Schoenmakers, P. J., Nanoparticle Analysis by Online Comprehensive Two-Dimensional Liquid Chromatography combining Hydrodynamic Chromatography and Size-Exclusion Chromatography with Intermediate Sample Transformation. *Anal. Chem.* 2017, 89, 9167–9174.

[31] Bedani, F., Kok, W. T., Janssen, H.-G., A theoretical basis for parameter selection and instrument design in comprehensive size-exclusion chromatography×liquid chromatography. *J. Chromatogr. A* 2006, 1133, 126–134.

[32] Popovici, S. T., Schoenmakers, P. J., Fast size-exclusion chromatography--theoretical and practical considerations. *J. Chromatogr. A* 2005, 1099, 92–102.

[33] Edam, R., Mes, E. P. C., Meunier, D. M., Van Damme, F. A., Schoenmakers, P. J., Branched polymers characterized by comprehensive two-dimensional separations with fully orthogonal mechanisms: Molecular-topology fractionation×size-exclusion chromatography. *J. Chromatogr. A* 2014, 1366, 54–64.

[34] Mondello, L., Tranchida, P. Q., Stanek, V., Jandera, P., Dugo, G., Dugo, P.,

Silver-ion reversed-phase comprehensive two-dimensional liquid chromatography combined with mass spectrometric detection in lipidic food analysis. *J. Chromatogr. A* 2005, 1086, 91–98.

[35] Yang, Q., Shi, X., Gu, Q., Zhao, S., Shan, Y., Xu, G., On-line two dimensional liquid chromatography/mass spectrometry for the analysis of triacylglycerides in peanut oil and mouse tissue. *J. Chromatogr. B* 2012, 895–896, 48–55.

[36] Hu, J., Wei, F., Dong, X.-Y., Lv, X., Jiang, M.-L., Li, G.-M., Chen, H., Characterization and quantification of triacylglycerols in peanut oil by off-line comprehensive two-dimensional liquid chromatography coupled with atmospheric pressure chemical ionization mass spectrometry. *J. Sep. Sci.* 2013, 36, 288–300.

[37] Dugo, P., Kumm, T., Crupi, M. L., Cotroneo, A., Mondello, L., Comprehensive two-dimensional liquid chromatography combined with mass spectrometric detection in the analyses of triacylglycerols in natural lipidic matrixes. *J. Chromatogr. A* 2006, 1112, 269–275.

[38] Hu, L., Li, X., Feng, S., Kong, L., Su, X., Chen, X., Qin, F., Ye, M., Zou, H., Comprehensive two-dimensional HPLC to study the interaction of multiple components in Rheum palmatum L. with HSA by coupling a silica-bonded HSA column to a silica monolithic ODS column. *J. Sep. Sci.* 2006, 29, 881–888.

[39] François, I., Sandra, P., Comprehensive supercritical fluid chromatography × reversed phase liquid chromatography for the analysis of the fatty acids in fish oil. *J. Chromatogr. A* 2009, 1216, 4005–4012.

[40] Hirata, Y., Ozaki, F., Comprehensive two-dimensional capillary supercritical fluid chromatography in stop-flow mode with synchronized pressure programming. *Anal. Bioanal. Chem.* 2006, 384, 1479–1484.

[41] Pirok, B. W. J., Knip, J., van Bommel, M. R., Schoenmakers, P. J., Characterization of synthetic dyes by comprehensive two-dimensional liquid chromatography combining ion-exchange chromatography and fast ion-pair reversed-phase chromatography. *J. Chromatogr. A* 2016, 1436, 141–146.

[42] Pirok, B. W. J., Pous-Torres, S., Ortiz-Bolsico, C., Vivó-Truyols, G., Schoenmakers, P. J., Program for the interpretive optimization of two-dimensional resolution. *J. Chromatogr. A* 2016, 1450, 29–37.

[43] Snyder, L. R., Dolan, J. W., Gant, J. R., Gradient elution in high-performance liquid chromatography. *J. Chromatogr. A* 1979, 165, 3–30.

[44] Snyder, L. R., Poppe, H., Mechanism of solute retention in liquid—solid chromatography and the role of the mobile phase in affecting separation. *J. Chromatogr. A* 1980, 184, 363–413.

[45] Akaike, H., A New Look at the Statistical Model Identification. *IEEE Trans. Automat. Contr.* 1974, 19, 716–723.

[46] Česla, P., Vaňková, N., Křenková, J., Fischer, J., Comparison of isocratic retention models for hydrophilic interaction liquid chromatographic separation of native and fluorescently labeled oligosaccharides. *J. Chromatogr. A* 2016, 1438, 179–188.

[47] Schure, M. R., Quantification of resolution for two-dimensional separations. *J. Microcolumn Sep.* 1997, 9, 169–176.
